# Marine Sediment-Derived *Streptomyces* Bacteria from British Columbia, Canada Are a Promising Microbiota Resource for the Discovery of Antimicrobial Natural Products

**DOI:** 10.1371/journal.pone.0077078

**Published:** 2013-10-10

**Authors:** Doralyn S. Dalisay, David E. Williams, Xiao Ling Wang, Ryan Centko, Jessie Chen, Raymond J. Andersen

**Affiliations:** Departments of Chemistry and Earth, Ocean and Atmospheric Sciences, University of British Columbia, Vancouver, B.C., Canada,; University of East Anglia, United Kingdom

## Abstract

Representatives of the genus *Streptomyces* from terrestrial sources have been the focus of intensive research for the last four decades because of their prolific production of chemically diverse and biologically important compounds. However, metabolite research from this ecological niche had declined significantly in the past years because of the rediscovery of the same bioactive compounds and redundancy of the sample strains. More recently, a new picture has begun to emerge in which marine-derived *Streptomyces* bacteria have become the latest hot spot as new source for unique and biologically active compounds. Here, we investigated the marine sediments collected in the temperate cold waters from British Columbia, Canada as a valuable source for new groups of marine-derived *Streptomyces* with antimicrobial activities. We performed culture dependent isolation from 49 marine sediments samples and obtained 186 *Streptomyces* isolates, 47 of which exhibited antimicrobial activities. Phylogenetic analyses of the active isolates resulted in the identification of four different clusters of bioactive *Streptomyces* including a cluster with isolates that appear to represent novel species. Moreover, we explored whether these marine-derived *Streptomyces* produce new secondary metabolites with antimicrobial properties. Chemical analyses revealed structurally diverse secondary metabolites, including four new antibacterial novobiocin analogues. We conducted structure-activity relationships (SAR) studies of these novobiocin analogues against methicillin-resistant *Staphylococcus aureus* (MRSA). In this study, we revealed the importance of carbamoyl and OMe moieties at positions 3” and 4” of novobiose as well as the hydrogen substituent at position 5 of hydroxybenzoate ring for the anti-MRSA activity. Changes in the substituents at these positions dramatically impede or completely eliminate the inhibitory activity of novobiocins against MRSA.

## Introduction

The discovery of new bioactive natural products from marine sources has become an important research area due to the extraordinary chemical diversity and novelty found in marine natural products and their potential applications as lead compounds in drug development [1,2]. The ocean covers approximately 70% of the Earth’s surface and has the potential to provide a treasure trove of largely unexplored biodiversity. In the past 30 years, bioprospecting for new marine natural products has yielded several thousand chemically diverse compounds [3]; in 2009 to 2010 alone 2,014 novel marine natural products were discovered [4,5]. The rate of discovery is expected to escalate with the advancement of emerging technological tools, dedicated research programs, and most importantly, collaborative research efforts amongst microbiologists, molecular biologists, chemical biologists, organic chemists, biochemists, bioengineers, pharmacologists and ecologists[1]. 

Interest in marine microorganisms as a source for natural products has grown considerably in the past decade[1,6,7]. The world’s oceans provide the largest habitat for microorganisms on earth and they are the home to diverse uncharacterized microbial communities. Marine microorganisms represent a vast untapped resource of novel biologically active natural products. In 2010 more than 300 structurally diverse bioactive natural products were discovered from marine microorganisms and phytoplankton species [4,5]. Clearly, such recent discoveries suggest that marine microorganisms have the potential to be a massive resource for bioactive natural product discovery.

Members of the genus *Streptomyces* are ubiquitous soil actinomycetes and a proven source of bioactive secondary metabolites, particularly antibiotics for medical, agricultural and veterinary use [8]. These bioactive secondary metabolites represent an array of different and structurally diverse chemical classes such as polyketides, peptides, macrolides, indoles, aminoglycosides and terpenes [4,5,9,10]. Although their ecological role in marine environments and biogeographical distribution remains unknown, reports have shown that actinomycetes are widely distributed in the oceans, even in the deepest trenches [11]. The majority of antibiotic-producing *Streptomyces* strains have been isolated from terrestrial environments, while those from the marine environment have been largely ignored [10,12]. However, in recent years marine sediments have become a geographical hot spot for sourcing and cultivation of *Streptomyces* strains. This sampling strategy has resulted in the discovery of a new taxon of marine actinomycetes, *Salinispora* [12]. These obligate marine and metabolically rich bacteria produce a multitude of interesting natural products of biological importance. Salinosporamide A, a γ-lactam-β-lactone inhibitor of the 20S proteasome [13], was isolated from *S. tropica* [14] and has entered phase 1 clinical trials to treat patients with multiple myeloma, solid tumors, and lymphoma [13,15]. Aside from salinosporamide A [10,15], diazepinomicin has entered clinical investigation for its efficacy observed against advanced cancer and is an investigational drug being developed by Thallion Pharmaceuticals Inc. [16]. Diazepinomicin is an alkaloid isolated from marine-derived *Micromonospora* sp. DPJ12 [17], and is a potent inducer of apoptosis binding to the benzodiazepine receptor and inhibiting the Ras/MAP kinase pathway [16]. Another promising compound is thiocoraline, a thiodepsipeptide isolated from marine-derived *Micromonospora marina* [18], that disrupts DNA elongation by inhibiting DNA polymerase alpha at concentrations that inhibit cell cycle progression [19]. The investigation of this compound as a potential drug lead is currently being conducted at PharmaMar, where thiocoraline derivatives are undergoing pre-clinical trials [20]. The discovery of these compounds, from only a tiny subset of the marine microbiota, with unique structural features and molecular modes of action underscores the importance of novel genera of marine-derived actinomycetes as sources of unprecedented bioactive secondary metabolites and as leads for drug discovery. 

Despite the recent success of bioactive natural products discovery from marine-derived microorganisms, there is an urgent need for additional novel drug-like compounds, especially antibiotics. Reports identify a rising incidence of multidrug-resistant pathogenic bacteria that infect people of all ages and health states [21]. In addition, another cause of concern is that in the past decade only four antibiotics belonging to new structural classes have been approved for clinical use [22]. While most of the published reports on bioactive marine-derived actinomycetes were isolated from tropical and sub-tropical marine sediments [10,12], no reports have been published on the *Streptomyces* bacterial community cultivated from sediments collected in cold/temperate waters of the Northeast Pacific Ocean. 

In response to the challenge and crisis of widespread antibiotic resistance, we have examined marine sediments collected in the cold/temperate waters of Georgia Strait, Bamfield, Howe Sound, and Indian Arm, British Columbia, Canada as a source for new groups of marine-derived *Streptomyces* with antimicrobial activities. Specifically, we aimed to evaluate the diversity of the *Streptomyces* community and isolate *Streptomyces* cultivated from marine sediments by a series of selective media dependent experiments. This has resulted in the identification of four different clusters of bioactive *Streptomyces* including a cluster with isolates that may appear to represent novel species. In addition, we also aimed to explore whether these marine-derived *Streptomyces* produce new secondary metabolites with antimicrobial properties. Chemical characterization of the bioactive compounds revealed structurally diverse secondary metabolites, including four new novobiocin analogues. In addition, we evaluated the structure-activity-relationship (SAR) implications of the new novobiocin analogues against methicillin resistant *Staphylococcus aureus* (MRSA) and identified key structural features required to elicit anti-MRSA activity. 

## Results and Discussion

A total of 186 *Streptomyces* bacteria were isolated from 49 sediment samples collected from a range of locations and depths (20 to 200 m) in British Columbian waters from 2007 to 2009 (Table 1 and Table S1 in File S1) (see Materials and Methods). *Streptomyces* were most prevalent in the Georgia Strait samples, collected at a depth of 70 to 190 m. These yielded the highest total percentage of cultivatable *Streptomyces* (47%, 88 isolates), using different selective media, followed by Indian Arm fjord samples with a total percentage of 32% (60 isolates), and Bamfield having the lowest percentage of cultivatable *Streptomyces* (6%, 11 isolates). 

**Table 1:** Sample description.

**Collection site,^a^ year**	**Depth, m**	**No. of sediment samples**	**No. of *Streptomyces* isolated**	**No. of bioactive *Streptomyces* (%)**
Bamfield, 2007	60-150	9	11	6 (54)
Indian Arm, 2009	20-200	20	60	8 (13)
Georgia Strait, 2008	70-190	12	88	28 (32)
Howe Sound, 2009	80-125	8	27	5 (19)
Total			186	47 (25%)

a No specific permits were required for these field studies.

From the 28 selective media used for cultivating *Streptomyces*, two media yielded good recovery (Table 2): MM5 (44, 24%) and MM47 (42, 23%) which contained ISP4 and humic acid, respectively. The high yield rate from these media is not surprising because these organic substrates are the classic nutrients in cultivating *Streptomyces* [23]. Interestingly, MM17, which contained low concentrations of trehalose and soytone as organic substrates, yielded the third highest recovery - 18 (10%). Several combinations of organic substrates were tested, however, none of these yielded dramatic improvement on the culturability of the *Streptomyces* (Table 2). The mycelia of the isolates were observed after 7 to 15 days of incubation at room temperature. The aerial and substrate mycelia varied in color as did the soluble pigmentation of these isolates (Table S2 in File S1). All of the isolates tolerated salt concentration up to 3.5% but did not require salt for growth, indicating that these isolates were not obligate marine *Streptomyces*. This result suggests that these representative isolates may belong to common soil-inhabiting actinomycetes that have been washed from land and have adapted to the environment in marine sediments as dormant spores [10]. In addition, the 16S rRNA gene sequences of most of the isolates showed 100% identity to *Streptomyces* species isolated from land (Table 3). The presence of soil-derived *Streptomyces* in sediment samples collected from Indian Arm, Howe Sound and Georgia Strait was anticipated because of the proximity of the collection sites to large river run off. On the other hand, the absence of obligate marine *Streptomyces* from Bamfield sediment samples was fairly surprising because the collection sites were at the Pacific Ocean and away from river run off (e. g. N48 50.536 W125 13.028, depth 65 m and N48 52.830 W125 09.838, depth 82 m). This finding is provisionally explained by the difficulty to recover and cultivate obligate marine *Streptomyces* from sediments collected in cold/temperate waters of the Northeast Pacific Ocean. The processing method such as desiccation and heat shock [12,59] and incubation at 25 to 28°C used in this study may not apply well to recover obligate marine *Streptomyces*, therefore improved methodology and appropriate isolation techniques (e. g. incubation at 4 to 10 °C) possibly could recover and cultivate obligate marine *Streptomyces* from cold/temperate waters.

**Table 2:** *Streptomyces* recovery using various isolation marine media (MM).

**Medium**	**No. of *Streptomyces* isolated**	**Medium**	**No. of *Streptomyces* isolated**
1 MM1	9	15 MM18	1
2 MM3	2	16 MM19	2
3 MM5	44	17 MM21	3
4 MM6	1	18 MM22	3
5 MM7	1	19 MM23	2
6 MM8	5	20 MM24	2
7 MM10	6	21 MM25	2
8 MM11	6	22 MM28	3
9 MM12	1	23 MM38	5
10 MM13	2	24 MM39	2
11 MM14	4	25 MM47	42
12 MM15	1	26 MM48	6
13 MM16	1	27 MM49	4
14 MM17	18	28 MM50	8

**Table 3:** Marine sediment collection site, *Streptomyces* isolates, NCBI closest type strain and source of the nearest type strain.

**Collection site**	**Strain (accession #)**	**Most closely related type strain**	**16S rRNA gene sequence accession no.^a^**	**Sequence identity (%)**	**Source of nearest type strain^b^**
Bamfield	RJA2895 (JX535235)	*S. flavofungini* Szabo strain	EF571003	100	Lab strain
	RJA2910 (JX535236)	*S. hawaiiensis* NRRL 15010	EU624140	98.9	Soil
	RJA2921 (JX535237)	*S. deccanesis* DAS-139T	NR044183	98.0	Soil
	RJA2926 (JX535238)	*S. sporoclivatus* LMG 20312	AJ781369	99.9	Soil
	RJA3074 (JX535241)	*S. griseus* strain 52-1	EF571001	99.9	Soil
	RJA3265	*S. sampsonii* ATCC 25495	NR025870	99.7	Potato scab
Georgia Strait	RJA3937	*S. rutgersensis* DSM 40077T	Z76688	100	soil
	RJA3939	*S. flavofungini* Szabo strain	EF571003	100	Lab strain
	RJA3948(JX535242)	*S. koyangensis* VK-A60	NR025662	99.4	soil
	RJA3953	*S. sampsonii* ATCC 25495	NR025870	99.7	Potato scab
	RJA3956	*S. rutgersensis* DSM 40077T	Z76688	100	soil
	RJA3957	*S. rutgersensis* DSM 40077T	Z76688	100	soil
	RJA3958	*S. flavofungini* Szabo strain	EF571003	100	Lab strain
	RJA3972 (JF837443)	*S. rubrogriseus* LMG20318	AJ781373	99.3	Soil
	RJA3973	*S. flavofungini* Szabo strain	EF571003	100	Lab strain
	RJA3974	*S. flavofungini* Szabo strain	EF571003	100	Lab strain
	RJA3980	*S. rutgersensis* DSM 40077T	Z76688	100	Soil
	RJA3983 (JX535243)	*S. violaceusniger* Tu 4113	CP002994	99.8	Soil
	RJA3990 (JF837445)	*S. speibonae* PK-Blue	NR025212	98.6	Soil
	RJA3995	*S. sampsonii* ATCC 25495	NR025870	99.7	Potato scab
	RJA3996	*S. flavofungini* Szabo strain	EF571003	100	Lab strain
	RJA3999	*S. sampsonii* ATCC 25495	NR025870	99.7	Potato scab
	RJA4019	*S. flavofungini* Szabo strain	EF571003	100	Lab strain
	RJA4020 (JF510466)	*S. caeruleus* QD13II	EU274353	99.6	Soil
	RJA4028	*S. sampsonii* ATCC 25495	NR025870	99.7	Potato scab
	RJA4037	*S. sampsonii* ATCC 25495	NR025870	99.7	Potato scab
	RJA4038(JX535244)	*S. flocculus* NBRC 13041	NR041100	98.6	Soil
	RJA4040	*S. rutgersensis* DSM 40077T	Z76688	100	Soil
	RJA4053	*S. flavofungini* Szabo strain	EF571003	99.8	Lab strain
	RJA4054	*S. sampsonii* ATCC 25495	NR025870	99.7	Potato scab
	RJA4055(JX535245)	*S. drozdowiczii* NRRL B-24297	EF654097	98.6	Soil
	RJA4056	*S. rutgersensis* DSM 40077T	Z76688	100	Soil
	RJA4060	*S. sampsonii* ATCC 25495	NR025870	99.7	Potato scab
	RJA4081	*S. flavofungini* Szabo strain	EF571003	100	Lab strain
Howe Sound	RJA4068 (JX535246)	*S. anulatus* strain Malaysia	EU647478	99.5	Soil
	RJA4077	*S. flavofungini* Szabo strain	EF571003	100	Lab strain
	RJA4079	*S. flavofungini* Szabo strain	EF571003	100	Lab strain
	RJA3407	*S. flavofungini* Szabo strain	EF571003	100	Lab strain
	RJA3410	*S. flavofungini* Szabo strain	EF571003	100	Lab strain
Indian Arm	RJA2960	*S. flavofungini* Szabo strain	EF571003	100	Lab strain
	RJA2961 (JF719041)	*S. caeruleus* QD13II	EU274353	99.7	Soil
	RJA2969 (JX535239)	*S. sampsonii* ATCC 25495	NR025870	99.7	Potato scab
	RJA3024	*S. flavofungini* Szabo strain	EF571003	100	Lab strain
	RJA3025(JX535240)	*S. rutgersensis* DSM 40077T	Z76688	100	soil
	RJA3040	*S. sampsonii* ATCC 25495	NR025870	99.7	Potato scab
	RJA3067	*S. rutgersensis* DSM 40077T	Z76688	100	Soil
	RJA3622	*S. sampsonii* ATCC 25495	NR025870	99.7	Potato scab

a NCBI accession number.

b The corresponding type of strain had a level of similarity greater than or equal to 98.5%.

The 186 *Streptomyces* isolates were grown in nutrient rich marine medium agar, MM1 to assess their antimicrobial activities using a disc diffusion assay. After 14 days of incubation at room temperature, the biomass and medium were extracted with ethyl acetate (EtOAc) and the organic extracts were tested against six microorganisms: methicillin-resistant *Staphylococcus aureus*, MRSA (ATCC 33591), *Bacillus subtilis* (H344), *Escherichia coli* (UBC 8161), *Pseudomonas aeruginosa* (ATCC 27853), *Mycobacterium fortuitum* (ATCC 6842) and *Candida albicans* (ATCC 90028). Out of 186 *Streptomyces* extracts tested 47 (25%) exhibited bioactivity. Thirty-four (18%) of these extracts showed activity against *C. albicans*; 13 (7%) showed activity against MRSA; 3 (2%) were active against *B. subtilis* and one extract was active against *E. coli* (RJA4020), *P. aeruginosa* (RJA4020) and *M. fortuitum* (RJA2960). Interestingly, 5 extracts (RJA2960, RJA3957, RJA3983, RJA4020 and RJA4054) showed activity against two or more of the target microorganisms (Table 4). 

**Table 4:** Antimicrobial activities of *Streptomyces* isolates.

**Strain**	**Antimicrobial activities^a^**					
	***S. aureus*** ^b^ (ATCC 33591)	***B. subtilis*** (H344)	***C. albicans*** (ATCC 90028)	***P. aeruginosa*** (ATCC 27853)	***E. coli*** (UBC8161)	***M. fortiutum*** (ATCC 6842)
RJA2895			+			
RJA2910			+			
RJA2921	+					
RJA2926	+					
RJA2960			+			+
RJA2961	+					
RJA2969			+			
RJA3024			+			
RJA3025			+			
RJA3040			+			
RJA3067			+			
RJA3074			+			
RJA3265			+			
RJA3407			+			
RJA3410			+			
RJA3622			+			
RJA3937			+			
RJA3939			+			
RJA3948			+			
RJA3953			+			
RJA3956			+			
RJA3957	+	+				
RJA3958			+			
RJA3972	+					
RJA3973	+					
RJA3974	+					
RJA3980			+			
RJA3983		+	+			
RJA3990			+			
RJA3995			+			
RJA3996			+			
RJA3999			+			
RJA4019			+			
RJA4020	+			+	+	
RJA4028			+			
RJA4037			+			
RJA4038			+			
RJA4040	+					
RJA4053	+					
RJA4054	+	+				
RJA4055	+					
RJA4056			+			
RJA4060			+			
RJA4068	+					
RJA4077			+			
RJA4079			+			
RJA4081			+			

a Antimicrobial activity assay was performed by disk diffusion.

b Methicillin- resistant strain of *Staphylococcus aureus*

+ biologically active; has inhibitory activity

**Figure 1:**
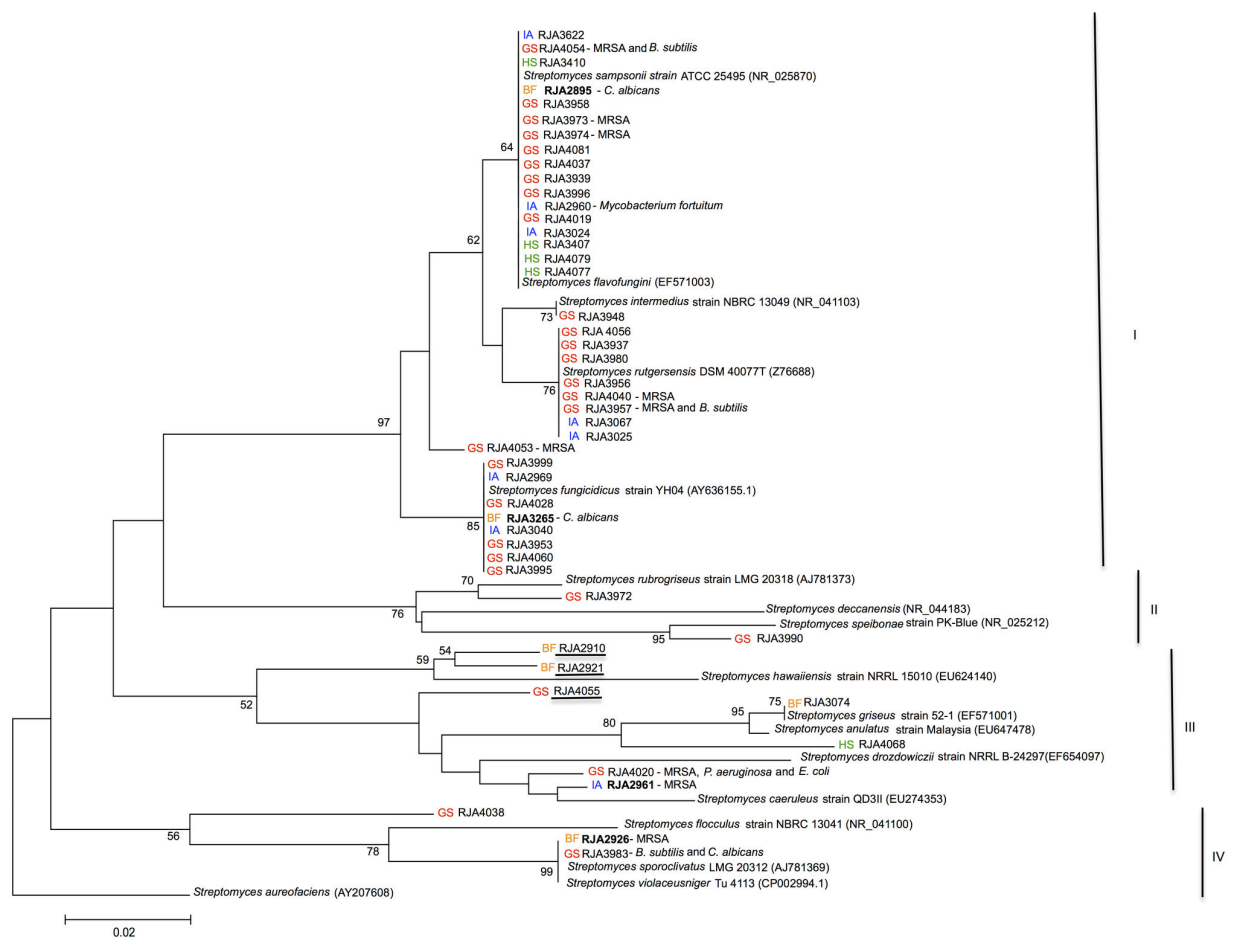
Phylogenetic tree constructed from partial 16S rRNA molecule that contains the highly variable γ-region. The evolutionary history was inferred using the Neighbor-Joining method [62]. The optimal tree with the sum of branch length = 0.86861675 is shown. The percentages of replicate trees in which the associated taxa clustered together in the bootstrap test (2000 replicates) are shown next to the branches with values higher than 50%. *Streptomyces aureofaciens* (AY207608) served as an outgroup. The tree is drawn to scale, with branch lengths in the same units as those of the evolutionary distances used to infer the phylogenetic tree. The evolutionary distances were computed using the Jukes-Cantor method [63] and are in the units of the number of base substitutions per site. All positions containing gaps and missing data were eliminated. There were a total of 115 positions in the final dataset. Evolutionary analyses were conducted in MEGA5 [64]. The scale bar represents 0.02 substitutions per nucleotide position. GS, Georgia Strait; IA, Indian Arm; BF, Bamfield; HS, Howe Sound. *Streptomyces* strains that were chemically analyzed are highlighted in bold and those that may represent new species are underlined. National Center for Biotechnology Information (NCBI) accession numbers are given next to the strain name.

The 16S rRNA gene sequences of the 47 active *Streptomyces* isolates were examined by phylogenetic analysis. Comparison and BLAST searches of the nearly complete 16S rRNA gene sequences (averaging 1,440 nucleotides) verified that these isolates were closely or completely identical (98 to 100%) to reported *Streptomyces* in the Genbank database. The 120 base pair nucleotide sequence of the 16S rRNA molecule that contains the highly variable γ-region to classify representatives and resolve relationship within the genus *Streptomyces* [24] was used to construct a multiple alignment and a phylogenetic tree (Figure 1). This tree of 47 active *Streptomyces* revealed 4 major phylogenetic clusters on the basis of bootstrap values (97, 76, 52 and 56). Cluster I formed multiple phyletic lines, which is supported by 97% of bootstrap replicates. The cluster includes groups, which are closely related to soil-derived *Streptomyces*: *S. flavofungini* (EF571003), *S. sampsonii* (NR_025870), *S. rutgersensis* (Z76688) and *S. fungicidicus* (AY636155). The 16S rRNA gene sequences of fourteen isolates were 100% identical to *S. flavofungini*, 3 were nearly identical to *S. sampsonii* (99.7%) and 8 showed 100% identity with *S. fungicidicus*. Representatives of this cluster were isolated from marine sediments collected from Georgia Strait, Indian Arm, Bamfield and Howe Sound. The organic extracts of almost all representative isolates in cluster I showed activity against *C. albicans*. Interestingly, from the fourteen isolates showing high similarity to *S. flavofungini*, two isolates, RJA3973 and RJA 3974 demonstrated activities against only MRSA. In addition, only one isolate, RJA2960 showed activity against *M. fortuitum* and *C. albicans*. The same result was observed among the three isolates showing high similarity to *S. sampsonii* where one isolate, RJA4054, was active against MRSA and *B. subtilis* but not *C. albicans*. Isolates RJA3025, RJA3067, RJA3937, RJA3956, RJA3957, RJA3980, RJA4040 and RJA4056, which shared 100% identity to soil-derived *S. rutgersensis* and RJA 3948 with 99.9% 16S rRNA gene sequence identity to *S. intermedius* (NR_041103) belong to two phyletic lines supported by 76% and 73% bootstrap replicates, respectively. Interestingly, all of these representative isolates showed activity against *C. albicans*, except for two isolates RJA4040 and RJA3957. Isolate RJA4040 demonstrated activity against just MRSA while isolate RJA3957 showed activity against MRSA and *B. subtilis*. The antibiotic phenotype differences observed among phylogenetically related *Streptomyces* species in this cluster is most likely due to the absence of specific antibiotic genes, presence of diverse biosynthetic genes or differences in regulation of gene expression among these related species [25-27].

Cluster II formed two phyletic lines, supported by 76 and 70% bootstrap replicates. Only two isolates belong to this cluster: Isolate RJA3990 which had a 16S rRNA gene sequence 98.6% identical to a soil-derived *S. speibonae* (NR_025212) (98.6%) and demonstrated activity against *C. albicans*; and RJA 3972 which showed 99.3% gene sequence identity to a soil-derived *S. rubrogriseus* (AJ781373). Both of these representative isolates were from Georgia Strait sediment samples. 

Cluster III was comprised of seven representative isolates. Three of the isolates where from Bamfield, two from Georgia Strait, one from Howe Sound and one from Indian Arm. Isolate RJA2910 had 98.9% 16S rRNA gene sequence identical to *S. hawaiiensis* (EU624140) and was active against *C. albicans*. Another isolate in this cluster RJA2921, showed activity against MRSA and shared 98.0% 16S rRNA sequence identity to soil-derived *S. deccanesis* (NR_044183). Isolates RJA 2961 and RJA4020 showed 99.7 and 99.6% sequence identities, respectively, to *S. caeruleus* (EU274353). Both of the isolates demonstrated activity against MRSA, however RJA4020 was also active against *E. coli* and *P. aeruginosa*, making RJA4020 the only representative isolate with Gram-negative activity. Isolate RJA4055 shared a 16S rRNA gene sequence identity of 98.6% to a soil-derived *S. drozdowiczii* (EF654097) and this isolate was active against MRSA. Another representative isolate was RJA4068, which shared 99.5% identity to the 16S rRNA gene sequence of *S. anulatus* (EU647478) and demonstrated activity against MRSA. The last representative, RJA3074 shared 99.9% identity to the 16S rRNA gene sequence of soil-derived *S. griseus* (EF571001) and it exhibited activity against *C. albicans*. It is interesting to note that five out of seven representative isolates in cluster III had activity against MRSA. Most of the *Streptomyces* isolates from this cluster had 99.6 to 99.9% 16S rRNA nucleotide sequence identity to their related species in the GenBank database, indicating that these are isolates of established *Streptomyces* species. Interestingly, isolates RJA2910, RJA2921 and RJA 4055 shared 16S rRNA gene sequence identity of 98.0% to 98.6% to their related species in the GenBank database. These bioactive isolates may represent new species in this cluster, however, the coherence of cluster III as a well-defined taxon is weakened by its low bootstrap value (52%). Therefore, the phylogenetic position and bioactivity relationship for this cluster is unclear. Nonetheless, comprehensive characterization of these bioactive isolates using molecular and chemotaxonomic approaches is currently ongoing in our laboratory. 

Cluster IV showed similarity to cluster II. It also formed two phyletic lines, supported by 56 and 78% bootstrap replicates and comprised by three representative isolates from Georgia Strait, and Bamfield samples. Isolate RJA4038 that is active against *C. albicans*, shared 98.6% identity to its nearest neighbor in the cluster, soil-derived *S. flocculus* (NR_041100). This phylogenetic and bioactivity relationship is fairly poor due to low bootstrap value (56%). Isolates RJA2926 and RJA3983 form a monophyletic sub-cluster with *S. sporoclivatus* and *S. violaceusniger*, which is supported by high bootstrap value (99%). Isolate, RJA2926 had 99.9% sequence identity to *S. sporoclivatus* (AJ781369) and demonstrated activity against MRSA. The last isolate, RJA3983, which shared 99.8% gene sequence identity with soil-derived *S. violaceusniger* (CP002994) exhibited activity against to both *B. subtilis* and *C. albicans*. 

**Figure 2:**
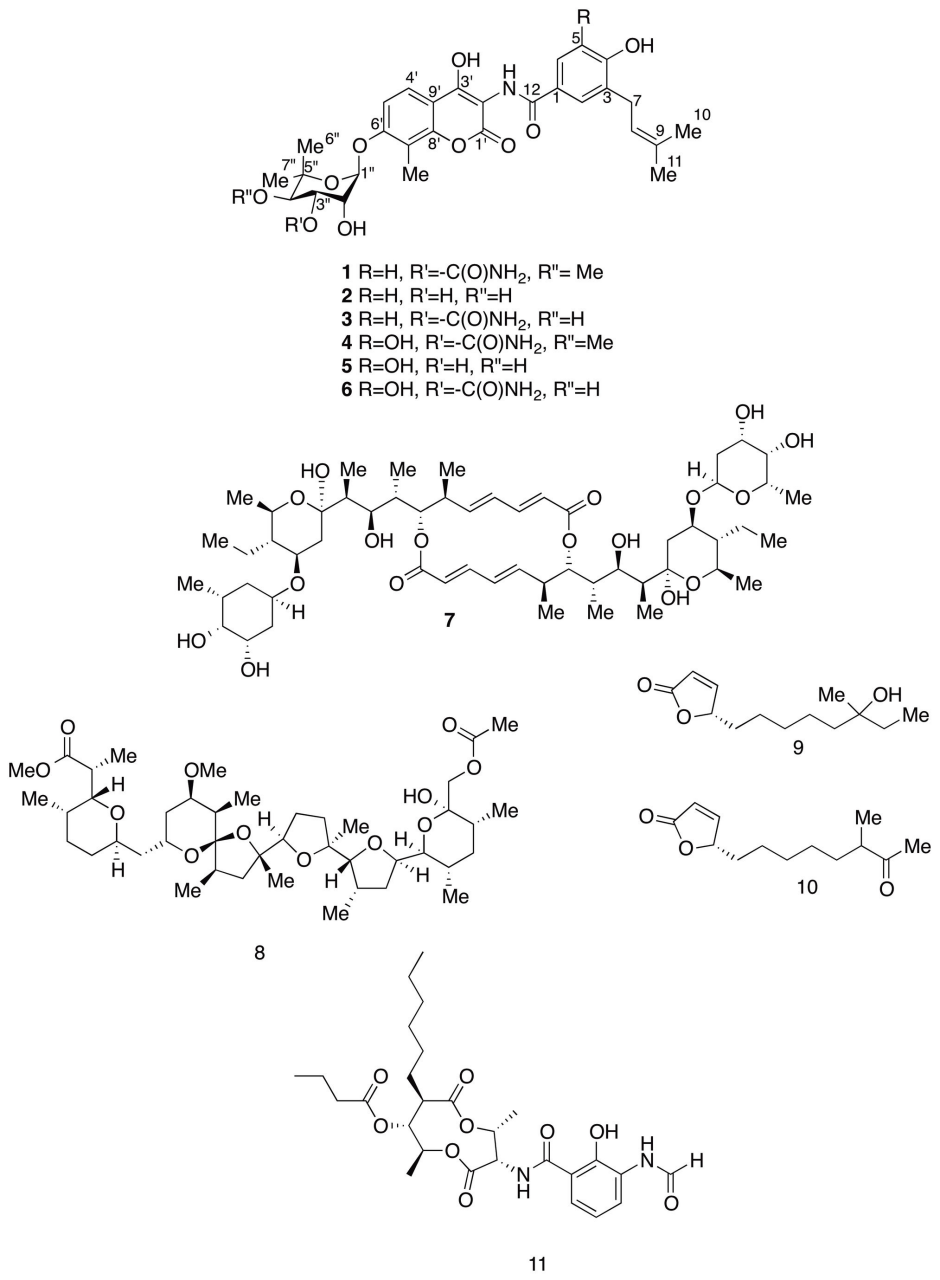
Structures of novobiocins (1-6), elaiophylin (7), nigericin (8), butanolides (9, 10) and antimycin A2B (11).

Of the 47 active *Streptomyces* isolates, we chemically analyzed the organic extracts of the four isolates (RJA2961, RJA2926, RJA2895 and RJA3265) that exhibited the strongest antimicrobial activity against the test organisms and possessed unique phenotypic characteristics (Table S2 in File S1). Production cultures of the producing organism were grown as lawns on solid agar MM1 at room temperature. In each case, the cells and media from the solid agar culture were extracted repeatedly with EtOAc. Concentration of the combined EtOAc extracts in vacuo gave a residue that was partitioned between EtOAc and H_2_O. Bioassay guided fractionation of the EtOAc-soluble material using sequential application of Sephadex LH20 chromatography and reversed-phase HPLC gave pure samples of the bioactive components for each of the four isolates. Four new novobiocins (**3-6**) (RJA2961) were isolated in addition to the known antibiotics - elaiophylin (**7**), and nigericin (**8**) both from RJA2926; butenolides (**9** and **10**) from RJA 2895; and antimycin A2B (**11**) from RJA 3265 (Figure 2). 

The anti-MRSA compounds isolated from RJA 2926 were known antibiotics, **7** and **8**. Elaiophylin (**7**), was first isolated from soil-derived *S. melanosporus* [28] and later from *S. violaceusniger* [29]. It is a macrolide with 16-membered unsaturated lactone ring possessing a characteristic C2 symmetry structure and has strong activity against Gram-positive bacteria [30]. Another compound isolated from the extract of RJA 2926 was nigericin, **8**. This compound was first isolated from soil-derived *S. hygroscopicus* [31,32] and has shown potent activity against Gram-positive bacteria [33]. Its mode of action was best explained by its cation complexation and as an ionophore in biological membranes [34]. The presence of **7** and **8** in the RJA 2926 extract provides a chemical explanation for the strong activity of the extract against MRSA (32 mm zone of inhibition at 40 μg/disc), which further suggests a synergistic effect of these two known antibiotics against MRSA. The antifungal compounds isolated from the organic extract of RJA2895 were known butenolides **9** [35] and **10** [36]. These known lactone-containing metabolites were isolated from a marine sediment derived *Streptomyces* species collected from Korea and North Atlantic Ocean (**9** and **10**, respectively) and were active against *C. albicans* [35,36]. Again, the co-occurence of butenolides **9** and **10** in RJA2895 extract explained the strong activity observed against *C. albicans* (37 mm zone of inhibition at 40 μg/disc). The major compound isolated from RJA 3265 was a known antifungal, antimycin A2B, **11** [37]. Antimycin A2B, belongs to the family of chemically labile nine-membered ring dilactonic antibiotics, the antimycins [38,39]. This family of antifungal compounds impede the respiratory chain by inhibiting the electron transfer of the ubiquinol:cytochrome c reductase (complex III) of the mitochondrial respiratory chain [40,41]. There were several analogues of antimycin observed from the RJA3265 extract by UV, NMR and HPLC profiles. The presence of these analogues provided the basis for the strong antifungal activity of RJA3265 against *C. albicans* (30 mm zone of inhibition at 40 μg/disc). 

Investigation of the organic extract from cultures of the *Streptomyces* strain RJA2961 resulted in the isolation of novobiocin **1**, desmethyldescarbamoylnovobiocin **2** and four new novobiocins analogues, compounds **3**-**6**. Details of the structure elucidation of these compounds are presented below. Although novobiocin was first reported in the mid-1950’s [42] it was not until 1999 that a complete NMR assignment was reported in the literature [43]. Our data was in complete agreement (except for the assignment of carbons C-12 and C-1’, which we feel should be switched) with that of Crow et al [43] (see Table S3 in File S1). With the structure of **1** established we could readily assign the structure of desmethyldescarbamoylnovobiocin **2** to a second peak isolated off the HPLC, since both the carbamoyl (δ 156.3/-302.6/6.73 bs, 6.52 bs) and OMe (δ 61.0/3.46 s) ^13^C, ^15^N and ^1^H NMR resonances were no longer present. These had been replaced by two additional OH doublet resonances in the ^1^H NMR spectrum at δ 4.79 (*J* = 5.4) and 4.96 (*J* = 5.4), that did not correlate to carbons or nitrogens in the gHSQC experiments, but showed gCOSY correlations to H-3” (δ 3.89) and H-4” (δ3.57), respectively. Although **2** is described in the literature as an intermediate in the biosynthetic pathway of **1** [44,45], to the best of our knowledge no NMR assignments have been presented in the scientific literature for the structure of **2** so these have been included in Table S3 in File S1. 

Desmethylnovobiocin 3 was isolated as pale yellow optically active amorphous solid that gave a [M + Na]^+^ ion in the HRESIMS at *m/z* 621.2078 appropriate for a molecular formula of C_30_H_34_N_2_O_11_, that differed from the molecular formula of **1** simply by the loss of CH_2_. As with desmethyldescarbamoylnovobiocin **2** the OMe resonances seen in the NMR spectra of **1** were no longer present and the carbon resonance assigned to C-4” (δ 80.7) in **1** was shifted upfield to δ 69.8. Assigning an OH at C-4” rather than the OMe substituent observed in **1** accounted for the loss of CH_2_ and structure **3** was assigned to desmethylnovobiocin.

5-Hydroxynovobiocin **4** gave a [M - H]^-^ ion in the HRESIMS at *m/z* 627.2175 appropriate for a molecular formula of C_31_H_36_N_2_O_12_, that differed from the molecular formula of **1** by the addition of oxygen. The ^1^H and ^13^C NMR spectra of **4** were markedly similar to those of **1** except that the two aromatic proton doublet resonances seen for **1** at δ 6.85 (*J* = 8.2) (H-5) and δ 7.71 (*J* = 8.2) (H-6) had been replaced by an aromatic singlet at δ 7.29 (H-6), that correlated to a carbon at δ113.1 in the gHSQC, and a singlet at δ 9.61 (5-OH) that did not correlate to carbon or nitrogen in the gHSQC experiments. In the gHMBC NMR the proton resonance at δ 9.61 (5-OH) correlated to three aromatic carbons at δ 146.7, 144.3, 113.1 that could be assigned to C-4, C-5 and C-6, respectively, when an OH substituent is placed at C-5. This evidence led us to assign structure 4 to 5-hydroxynovobiocin.

Measurement of the [M+Na]^+^ in the HRESIMS for desmethyldescarbamoyl-5-hydroxynovobiocin **5** and desmethyl-5-hydroxynovobiocin **6** gave the molecular formula for these two compounds as C_29_H_33_NO_11_ and C_30_H_34_N_2_O_12_, respectively. Analysis of the ^1^H/^13^C/gCOSY60/gHSQC/gHMBC/^15^NgHSQC/^15^NglrHMQC NMR data obtained for **5** and **6** (Table S3 in File S1) showed the same relationship between compounds **4**, **5** and **6** as seen previously for compounds **1**, **2** and **3**. As with desmethyldescarbamoylnovobiocin **2** both the carbamoyl and OMe NMR resonances seem for **4** had been replaced by two OH doublet resonances in the ^1^H NMR spectrum of **5**. In the NMR spectra of **6** an OH doublet (δ 5.14, *J* = 6.2 Hz) replaced the OMe resonances, and correlated in the gHMBC experiment to a carbon resonating at δ 69.8 assigned to C-4”. This evidence led us to assign structures **5** and **6** to desmethyldescarbamoyl-5-hydroxynovobiocin and desmethyl-5-hydroxynovobiocin, respectively.

Novobiocin **1** is an antibiotic known to target bacterial gyrase by inhibiting the ATP hydrolysis [46]. The mechanism of interaction is well characterized by X-ray crystallographic analysis and biochemical studies revealing that the binding of **1** to the β subunit of the DNA gyrase requires both the aminocoumarin and the noviose moieties of the antibiotic [47,48]. The carbamoyl group in **1** plays a critical role in forming hydrogen bonding between the antibiotic and ATP-binding site in the GyrB subunit. Moreover, **1** overlaps with the ATP binding site – the noviose moiety binds at the same place as the adenine ring of ATP which explains the high affinity of aminocoumarin to bacterial gyrase and thus establishing the structural basis for its competitive inhibitory nature. It was previously shown that **1** also interacts with the heat shock proteins (Hsp90) at the C-terminus of ATP-binding pocket causing destabilization of Hsp90 chaperon proteins [49]. This family of proteins is essential in eukaryotic cell signaling, proliferation and survival [50] and consequently has become a potential target in the treatment of cancer due to the critical role of the folding machinery of Hsp90 protein in the stability, refolding, and maturation of tumor cells [51]. Novobiocin **1** was licensed for clinical use to treat infections under the trade name Albamycin (Pharmacia and Upjohn) in the 1960’s but was later withdrawn from the market due to its high eukaryotic toxicities, insolubility in water, and low therapeutic activities against Gram-negative infections (resulting from poor permeability) [51]. More recently novobiocin in combination with rifampin has been found to be effective in treating MRSA colonization [52], with the added bonus of reducing the emergence of resistance. As would be expected there has been considerable interest in improving the potency and the selectivity of **1** both as a DNA gyrase inhibitor [51] and as selective inhibitor of Hsp90, thus several structure activity relationship (SAR) studies have been undertaken with this in mind [53,54].

We investigated the structure-activity relationship of compounds **1**-**6** against MRSA by minimum inhibitory concentration using microbroth dilution assay. The biological data revealed that substituents at positions 3” and 4” of the noviose moiety as well as position 5 of hydroxybenzoate ring were essential for inhibitory activity. Changing the carbamoyl moiety at position 3” of noviose and the OMe substituent at position 4” eliminated the inhibitory activity as displayed by compounds **2** and **5** which bear OH groups at positions 3” and 4” of the noviose (Table 5). This observation confirms to the previous reports on the importance of carbamoyl moiety in forming hydrogen bonding at the ATP-binding site of the bacterial gyrase [47]. Compounds **2** and **5** may form weaker hydrogen bonding at this site resulting in the loss activity. This finding is not surprising, it was shown in a previous report that novobiocin analogues produced by recombinant techniques with 3”-OH in noviose displayed poor activity against *Bacillus subtilis* [53]. Interestingly, a synthetic analogue of novobiocin bearing OH group at position 3” of noviose instead of carbamoyl moiety has been shown to display high inhibitory activity against Hsp90 suggesting that the dual inhibitory activity of novobiocins against GyrB and Hsp90 is dependent on the substitution at position 3” of the noviose [54,55]. Novobiocin **1** and compound **4** have almost the same structural framework except at position 5 of the hydroxybenzoate ring. It is intriguing to note that a replacement of proton with a hydroxyl group at this position lowers the activity of **4** by 32 fold relative to **1** (Table 5), suggesting that replacement of OH at this position may affect the binding affinity of novobiocins to bacterial gyrase. Previous X-ray crystallography reports have shown that prenylated 4-hydroxybenzoyl moiety of aminocoumarin antibiotics has an important role in binding of the antibiotic to the gyrase in a mechanism that reduces the hydrophobic surface of the antibiotic as it wraps around the Pro74 of gyrase and folds back away from the solvent onto the coumarin ring leading to an increased interaction of the antibiotic and the bacterial gyrase [56]. Compound **3**, which only differs from **1** at position 4” of the noviose moiety showed significant decrease in its inhibitory activity. Replacement of OMe group to OH group at position 4”of the noviose moiety decrease drastically the MIC by 64 fold in comparison with **1** (Table 5); this implies that 4”-OMe moiety is important for the activity against MRSA. Previously published X-ray crystallographic data revealed that the extensive hydrogen bonding network between GyrB and novobiocin is contributed by not only by the 3”-carbamoyl moiety but also the 2”-OH and 4”-OMe moieties of the novoise ring [47]. Specifically, 4”-OMe moiety forms a hydrogen bond with the Asn46 side chain of GyrB [47]. This interaction may explain the decrease in MIC of compound **3** against MRSA and suggests that the replacement of OMe group at position 4” of the noviose moiety with an OH group may weaken the interaction of **3** to the target site. Although the 3”-carbamoyl moiety of the novoise is crucial in the formation of hydrogen bonding network in the GyrB binding site, a change of the substituents at positions 4” of novoise and position 5 of hydroxybenzoate ring as depicted in compound **6** produces analogs devoid of inhibitory activity against MRSA. 

**Table 5:** Minimum Inhibitory Concentration, MIC of novobiocins (1-6) against methicillin-resistant *Staphylococcus aureus*, MRSA (ATCC 33591).

Compound	MIC, μg/mL
1	0.25
2	>64
3	16
4	8
5	>64
6	>64

In conclusion, the combined approach of phylogenetic and chemical analyses adopted in this study show that the community of *Streptomyces* from marine sediments of British Columbia, Canada is a highly prolific resource of antimicrobial natural products for bioprospecting. Although, this work only reported the chemical analysis of a subset of the *Streptomyces* we cultivated, we are continuing our investigations of other promising isolates that have the potential to produce novel compounds with unique biological function[57,58]. Moreover, we found new antibacterial novobiocin analogues and have identified important features of the structure-activity relationship for these compounds against MRSA. In this study, we discovered that analogues bearing different substituents at 3”-carbamoyl and 4”-OMe noviose moieties, or a 5-H hydroxybenzoate ring had a dramatic decrease or complete elimination of inhibitory activity against MRSA. Our data provides useful insight for the antibiotic discovery process, specifically on the structure-activity relationship of novobiocins against MRSA.

## Materials and Methods

### Environmental sampling, processing and bacterial isolation

A total of 49 marine sediment samples were collected around the British Columbia coast and fjord from 2007 to 2009 (Table 1). No specific permits were required for these field studies. Sediments from depths of 20 to 60 m were collected by scuba diving, while sites at a depth of 80 to 200 m were obtained by a sand grabber, and the samples were placed in a sterile 50 mL conical tubes. Samples were kept at 4°C during sampling and were processed as soon as possible after collection by desiccation and heat shock method [12,59]. Samples were inoculated in marine media prepared with 100% natural sea water or normal saline solution (NSS) (17.60 g NaCl, 1.47 g of Na_2_SO_4_, 0.08 g NaHCO_3_, 0.25 g KCl, 0.04 g KBr, 1.87 g MgCl_2_.6H_2_O, 0.41 g of CaCl_2_.H_2_O, 0.008 g SrCl_2_.6H_2_O, 0.008 g H_3_BO_3_ and 1 L of deionized water) and amended with filtered (0.2 μm pore size) cycloheximide (100 μg/mL) and rifamycin (5 μg/mL), after autoclaving. The isolation media consist of the following: Marine medium 1 (MM1), 10 g of starch, 4 g of yeast extract, 2 g of peptone, 18 g of agar, and 1 L of natural sea water; MM3, 0.5 g of mannitol, 0.1 g of peptone, 15 g of agar, and 1 L of natural sea water; MM5, 37 g of ISP4, and 1 L of natural sea water; MM6, 0.5 g of cellobiose, 0.2 g of peptone, 15 g of agar, and 1 L of natural sea water; MM7, 2 g of sodium caseinate, 0.1 g of asparagine, 4 g of sodium propionate, 0.5 g of dipotassium phosphate, 0.1 g of MgSO_4_, 5 g of glycerol, 18 g of agar and 1 L natural sea water; MM8, 0.5 g of maltose, 0.2 g of peptone, 15 g of agar, and 1 L of NSS; MM10, 10 g soluble starch, 4 g yeast extract, 2 g peptone, 15 g of agar, and 1 L of natural sea water; MM11, 0.5 g of glucose, 0.5 g of yeast extract, 1 g of peptone, 0.01 g of FeSO_4_.7H_2_O, 0.02 g of Na_2_HPO_4_, 15 g of agar, and 1 L of natural sea water; MM12, 0.5 g of yeast extract, 0.2 g of beef extract, 0.5 g of peptone, 0.01 g of FeSO_4_.7H_2_0, 0.02 g of Na_2_HPO_4_, 15 g of agar, and 1 L of natural sea water; MM13, 0.5 g of trehalose, 0.2 g of peptone, 15 g of agar and 1 L of natural sea water; MM14, 0.5 g of sucrose, 0.2 g of peptone, 15 g of agar and 1 L of natural sea water; MM15, 0.5 g of xylose, 0.2 g of peptone, 15 g of agar and 1 L of natural sea water; MM16, 0.5 g of mannitol, 0.1 g of soytone, 15 g of agar and 1 L of natural sea water; MM17, 0.5 g of trehalose, 0.1 g of soytone, 15 g of agar and 1 L of natural sea water; MM18, 0.5 g of erythritol, 0.1 g of soytone, 15 g of agar and 1 L of natural sea water; MM19, 0.5 g of cellobiose, 0.1 g of soytone, 15 g of agar and 1 L of natural sea water; MM21, 8.37 g of MOPS 3-(N-morpholino)propanesulfonic acid, 0.07 g tricine, 0.01 g of FeSO_4_.7H_2_O, 2.3 g of K_2_HPO_4_, 0.5 g of NH_4_Cl_2_, 2 g of xylose and 1 L of NSS, MM22, 8.37 g of MOPS (3-(N-morpholino)propanesulfonic acid), 0.07 g tricine, 0.01g of FeSO_4_.7H_2_O, 2.3 g of K_2_HPO_4_, 0.5 g of NH_4_Cl_2_, 2 g of cellobiose and 1 L of NSS; MM23, 8.37 g of MOPS, 0.07 g tricine, 0.01 g of FeSO_4_.7H_2_0, 2.3 g of K_2_HPO_4_, 0.5 g of NH_4_Cl_2_, 2 g of erythritol and 1 L of NSS; MM24, 8.37 g of MOPS, 0.07 g tricine, 0.01 g of FeSO_4_.7H_2_0, 2.3 g of K_2_HPO_4_, 0.5 g of NH_4_Cl_2_, 2 g of maltose and 1 L of NSS; MM25, 8.37 g of MOPS 3-(N-morpholino)propanesulfonic acid, 0.07 g tricine, 0.01 g of FeSO_4_.7H_2_O, 2.3 g of K_2_HPO_4_, 0.5 g of NH_4_Cl_2_, 2 g of mannitol and 1 L of NSS; MM28, 8.37 g of MOPS, 0.07 g tricine, 0.01 g of FeSO_4_.7H_2_O, 2.3 g of K_2_HPO_4_, 0.5 g of NH_4_Cl_2_, 10 g of soluble starch and 1 L of NSS; MM38, 0.5 g of dextrose, 0.1 g of yeast extract, 0.1 g peptone, 8 g noble agar and 1 L of natural sea water, MM39, 0.5 g of dulcitol, 0.1 g of yeast extract, 0.1 g peptone, 10 g of Phytagel^TM^ and 1 L of natural sea water; MM47, 1 g of humic acid, 0.01 g of FeSO_4_.7H_2_O, 0.5 g of Na_2_HPO_4_, 1.71 g of KCl, 0.5 g of MgSO_4_.7H_2_O, 0.02 g CaCO_3,_ 5 mL of B vitamins (Sigma), 18 g of Agar and 1 L of natural sea water; MM48, 0.5 g of trehalose, 0.10 g of tryptose, 0.1 g of FeSO_4_.7H_2_O, 1.71 g of KCl, 0.10 of peptone, 10 g of Phytagel^TM^ and 1 L of natural sea water; MM49, 0.5 g of mannitol, 0.10 g of tryptose, 0.1 g of FeSO_4_.7H_2_O, 1.71 g of KCl, 0.10 of peptone, 10 g of Phytagel^TM^, 100 mL of NSS and 900 mL of natural sea water; MM50, 0.5 g of cellobiose, 0.1 g of peptone, 5 mL of B vitamins (Sigma), 10 g of Phytagel^TM^, 100 mL of NSS and 900 mL of natural sea water. The plates were incubated at room temperature (25 to 28°C) for 2 to 6 weeks and checked periodically for the growth of actinomycetes. Actinomycetes growing on the isolation plates were transferred with sterile inoculating loops to MM1 medium until they are pure. Pure cultures were stored frozen in MM1 broth (with no agar) with 20% glycerol at -80°C for long-term storage.

### Amplification, sequencing and analysis of 16S rRNA gene

The total DNA was isolated using a DNeasy blood and tissue kit (Qiagen) by picking a single of the pure cultured isolates according to the manufacturers instruction. Primers F27 (5’-AGAGTTTGATCCTGGCTCAG-3’) and RC_1492 (5’-TACGGCTACCTTGTTACGACTT’) were used to amplify 1490 bp of the 16S rRNA gene. The 50 μL of PCR mixture contained 5 μL total DNA isolated from different isolates (10 to 20 ng), 25 μL of iQ^TM^ SYBR® Green Supermix (Bio-Rad), 1 μL of F27 (10 μM), 1 μL of RC1492 (10 μM) and 18 μL Molecular Grade H_2_O. The PCR conditions were as follows: initial denaturation at 98°C for 3 min; 35 cycles at 98°C for 10 s, 60°C for 10s and 72°C for 60 s. The amplification products were cleaned up by QIAquick PCR cleanup kit (Qiagen) according to manufacturer’s protocol. The nearly complete 16S rRNA gene was sequenced by using the PCR products directly as sequencing templates with the following primers: F27 and RC192 (used in template amplification), R530 (5’-CCGCGGCTGCTGGCACGTA-3’) and F114 (5’-GCAACGAGCGCAACCC-3’) [12]. All sequencing reactions were carried out in Applied Biosystems 3730 DNA Sequencer at the Nucleic Acid Protein Service Units (NAPS), University of British Columbia. Comparison of the 16S rRNA gene sequences of the isolated actinomycete was determined by Basic Local Alignment Search Tool (nucleotide blast) [60] similarity searching GenBank nucleotide database. The 16S rRNA gene sequences of the related reference strains and other isolates were obtained from GenBank and aligned with ClustalX [61]. The phylogenetic tree based on the highly variable γ-region of the 16S rRNA gene sequence to classify representatives and resolve relationship within the genus *Streptomyces* [24] was used to construct a multiple alignment with the neighboring-joining method [62] and a matrix of Jukes-Cantor [63] distances provided with the Mega5 [64] software using 2,000 bootstrap replicates.

### Production and antimicrobial testing of isolates

The pure cultures were grown in MM1 agar with 0.001 g of KBr and FeSO_4_.7H_2_O (6 Petri dishes, 150 x 15mm: 400 mL of melted MM1 medium) for 10 to 14 days at room temperature (25 to 28°C). The mature culture was harvested by cutting the biomass and the medium in small squares, then transferred to a small plastic bucket and soaked in EtOAc (100 mL) for 24 hours before extraction. The crude extract was partitioned between H_2_O and EtOAc extract. The EtOAc layers was concentrated in vacuo and reconstituted in 100% dimethyl sulfoxide (25 mg/mL), and transferred to a 96-well plate for storage at -80°C until testing for antimicrobial activities. 

Antimicrobials assay were performed with the following test organisms: *Pseudomonas aeruginosa* (ATCC 27853), *Escherichia coli* (UBC 8161), Methicillin Resistant Staphylococcus aureus (MRSA) (ATCC33591), *Bacillus subtilis* (H344), *Candida albicans* (ATCC 90028), *Mycobacterium fortuitum* using disc diffusion assay. EtOAc extracts were delivered unto the sterile blank disc to make 40 μg/disc concentration. The zone of inhibition of the EtOAc extracts was measured in mm and compared to that of the positive controls - rifamycin (10 μg/disc), Polymyxin B (30 μg/disc), Ampothericin B (20 μg/disc) and negative control, DMSO.

### Minimun inhibitory concentration (MIC) assay

The in vitro susceptibility of each compound was determined by the broth microdilution method against methicillin-resistant *Staphyloccus aureus* (MRSA) ATCC33591. Briefly, 2-fold serial dilutions of compounds were prepared in 96-well microtiter plates from stock solutions in a Muller-Hinton broth medium to a final volume of 100 μL. The final drug concentrations tested were from 0.5 to 128 μg/mL. Bacterial inoculum was prepared from 24 h cultures on Tryptic Soy broth at 37 °C. The inoculum was diluted into Muller Hinton broth to yield a final inoculum with an optical density of 0.0001 (OD_600_). The microdilution wells, which contained 100 μL of the serially diluted compound, were inoculated with 100 μL of the resulting bacterial suspension. Four wells containing the drug-free medium, DMSO, and inoculum were used as controls. The inoculated plates were incubated at 37°C for 24 h. The growth was determined by measuring the OD at 600 nm using a DTX 880 (Beckman Coulter Inc.) plate reader. The MIC end point was defined as the lowest concentration with complete (90%) growth inhibition.

### Chemical analysis of culture extracts

The active isolates were cultured on 5 pans of solid agar, equivalent to 2 L volume of the marine medium 1 (10 g of soluble starch, 4 g of yeast extract, 2 g of peptone, 0.001 g of FeSO_4_.7H_2_O, 0.001 g of KBr, 18 g agar, 1 L sea water) at RT for 14 days. The mature cultures were sliced into small squares containing the bacterial biomass and the media, and extracted twice with EtOAc. The combined EtOAc extracts were concentrated in vacuo and partitioned between H_2_O and EtOAc. The EtOAc soluble material was chromatographed on Sephadex LH20 with 4:1 methanol/CH_2_Cl_2_ as eluent. The fractions were further purified to preparative reversed-phase HPLC to isolate the active pure compound. UV spectra were recorded with a Waters 996 Photodiode Array Detector. All solvents used for HPLC were Fisher HPLC grade. Merck Type 5554 silica gel plates and Whatman MKC18F plates were used for analytical thin layer chromatography. The ^1^H and ^13^C NMR spectra were recorded on a Bruker AV-600 spectrometer with a 5 mm CPTCI cryoprobe. ^1^H chemical shifts were referenced to the residual DMSO-*d*_6_, CD_2_Cl_2_ or acetone-*d*_6_ signal (δ 2.49, 5.32 and 2.05 ppm, respectively) and ^13^C chemical shifts are referenced to the DMSO-*d*_6_, CD_2_Cl_2_ or acetone-*d*_6_ solvent peak (δ 39.5, 54.0 and 206.7 ppm, respectively). Low and high resolution ESIQIT-MS were recorded on a Bruker-Hewlett Packard 1100 Esquire–LC system mass spectrometer. The resulting chemical data were compared with data from Antibase, Sci-Finder and MarinLit [65].

### Extraction and isolation of novobiocins 1-6

The solid agar cultures were extracted repeatedly with EtOAc. Concentration of the 4 L of combined EtOAc extracts in vacuo gave a gummy brown residue that was partitioned between EtOAc (3 x 150 mL) and H_2_O (300 mL). The combined EtOAc extract was evaporated to dryness and fractionated on Sephadex LH-20 using 4:1 MeOH/CH_2_Cl_2_ as eluent. The active fraction was then fractionated by C_18_ reversed-phase HPLC (CSC-Inertsil 150A/ODS2, 5 μm 25 x 0.94 cm column) with 1:1 MeCN/(0.05%TFA/H_2_O) as eluent to give pure samples of novobiocin (1) (13.3 mg) and 5-hydroxynovobicin (**4**) (6.1 mg), and mixtures of compounds **2** and **3**, and **5** and **6**. From the mixture of **2** and **3** using 43:57 MeCN/(0.05%TFA/H_2_O) as eluent, C_18_ reversed-phase HPLC gave a pure sample of desmethylnovobiocin (**3**) (6.7 mg) and after an additional HPLC step with 1:1 MeCN/(0.05%TFA/H_2_O) as eluent pure desmethyldescarbamoylnovobiocin (**2**) (1.1 mg) was obtained. Similarly from the mixture of **5** and **6** using 2:3 MeCN/(0.05%TFA/H_2_O) as eluent, C_18_ reversed-phase HPLC gave a pure sample of desmethyl-5-hydroxynovobiocin (**6**) (1.2 mg) and an additional HPLC step using the same eluent gave pure desmethyldescarbamoyl-5-hydroxynovobiocin (**5**) (0.4 mg). 

### Compound characterization

Novobiocin (**1**): Isolated as a pale yellow amorphous solid; [α]^25^D -40.3 (c 6.65, MeOH); UV (1:1 MeCN/(0.05%TFA/H_2_O) λ_max_ (log ε) 208 (4.0), 329 (3.7) nm; ^1^H, ^13^C and ^15^N NMR, see Table S3 in File S1; (+)-LRESIMS [M+Na]^+^ m/z 635.3 (calcd for C_31_H_36_N_2_O_11_Na, 635.2216).

Desmethyldescarbamoylnovobiocin (**2**): Isolated as a pale yellow oil; [α]^25^D -13.3 (c 0.55, MeOH); UV (57:43 (0.05%TFA/H_2_O)/MeCN) λ_max_ (log ε) 208 (3.8), 329 (3.5) nm; ^1^H (Figure S1 in File S1), ^13^C (Figure S2 in File S1) and ^15^N NMR, see Table S3 in File S1; (+)-HRESIMS [M+Na]^+^ m/z 578.1992 (calcd for C_29_H_33_NO_11_Na, 578.2002).

Desmethylnovobiocin (**3**): Isolated as pale yellow oil; [α]^25^D -33.6 (c 3.35, MeOH); UV (57:43 (0.05%TFA/H_2_O)/MeCN) λ_max_ (log ε) 208 (4.2), 329 (3.9) nm; ^1^H (Figure S3 in File S1), ^13^C (Figure S4 in File S1) and ^15^N NMR, see Table S3 in File S1; (+)-HRESIMS [M+Na]^+^ m/z 621.2078 (calcd for C_30_H_34_N_2_O_11_Na, 621.2060).

5-Hydroxynovobiocin (**4**): Isolated as a pale green amorphous solid; [α]^25^D -35.4 (c 3.05, MeOH); UV (1:1 MeCN/(0.05%TFA/H_2_O) λ_max_ (log ε) 208 (4.2), 329 (3.9) nm; ^1^H (Figure S5 in File S1), ^13^C (Figure S6 in File S1) and ^15^N NMR, see Table S3 in File S1; (-)-HRESIMS [M-H]^-^ m/z 627.2175 (calcd for C_31_H_35_N_2_O_12_, 627.2190).

Desmethyldescarbamoyl-5-hydroxynovobiocin (**5**): Isolated as a pale green amorphous solid; [α]25D 0.0 (c 0.26, MeOH); UV (3:2 (0.05%TFA/H_2_O)/MeCN) λ_max_ (log ε) 208 (4.0), 329 (3.7) nm; ^1^H (Figure S7 in File S1), ^13^C and ^15^N NMR, see Table S3 in File S1; (+)-HRESIMS [M+Na]^+^ m/z 594.1996 (calcd for C_29_H_33_NO_11_Na, 594.1951).

Desmethyl-5-hydroxynovobiocin (**6**): Isolated as a pale green amorphous solid; [α]^25^D -15.3 (c 0.60, MeOH); UV (3:2 (0.05%TFA/H_2_O)/MeCN) λ_max_ (log ε) 208 (4.1), 329 (3.8) nm; ^1^H (Figure S8 in File S1), ^13^C (Figure S9 in File S1) and ^15^N NMR, see Table S3 in File S1; (+)-HRESIMS [M+Na]^+^ m/z 637.2025 (calcd for C_30_H_34_N_2_O_12_Na, 637.2009).

### Nucleotide sequence accession numbers

The 16S rRNA sequence data reported in the present study were deposited in the Genbank nucleotide data base under the accession numbers JX535235 to JX535246, JF719041, JF837443, JF837445, JF510466

## Supporting Information

File S1 Table S1. List of *Streptomyces* isolates and coordinates of the collection site. Table S2. Descriptions of the aerial and substrate mycelia, and soluble pigment. Table S3. NMR Data for Novobiocins **1-6** (600 MHz, DMSO-*d*_6_). Figure S1: ^1^H NMR Spectrum of Desmethyldescarbamoylnovobiocin (**2**) recorded at 600 MHz in DMSO-*d*_6_. Figure S2: ^13^C NMR Spectrum of Desmethyldescarbamoylnovobiocin (**2**) recorded at 150 MHz in DMSO-*d*_6_. Figure S3: ^1^H NMR Spectrum of Desmethylnovobiocin (**3**) recorded at 600 MHz in DMSO-*d*_6_. Figure S4: ^13^C NMR Spectrum of Desmethylnovobiocin (**3**) recorded at 150 MHz in DMSO-*d*_6_. Figure S5: ^1^H NMR Spectrum of 4-Hydroxynovobiocin (**4**) recorded at 600 MHz in DMSO-*d*_6_. Figure S6: ^13^C NMR Spectrum of 4-Hydroxynovobiocin (**4**) recorded at 150 MHz in DMSO-*d*_6_. Figure S7: ^1^H NMR Spectrum of Desmethyldescarbamoyl-4-hydroxynovobiocin (**5**) recorded at 600 MHz in DMSO-*d*_6_. Figure S8: ^1^H NMR Spectrum of Desmethyl-4-hydroxynovobiocin (**6**) recorded at 600 MHz in DMSO-*d*_6_. Figure S9: ^13^C NMR Spectrum of Desmethyl-4-hydroxynovobiocin (**6**) recorded at 150 MHz in DMSO-*d*_6_.
(PDF)
