# Supplementary material for: Marine Sediment-Derived Streptomyces Bacteria from British Columbia, Canada Are a Promising Microbiota Resource for the Discovery of Antimicrobial Natural Products
Source: PLoS One. 2013 Oct 10;8(10):e77078. doi: 10.1371/journal.pone.0077078 (PMC3794959; doi:10.1371/journal.pone.0077078)

SUPPORTING INFORMATION

Marine sediment-derived *Streptomyces* bacteria from British Columbia, Canada are a promising microbiota resource for the discovery of antimicrobial natural products

Doralyn S. Dalisay[†‡], David E. Williams[†], Xiao Ling Wang[†], Ryan Centko[†], Jessie Chen[†], Raymond J. Andersen[†]*

[†]Departments of Chemistry and Earth, Ocean and Atmospheric Sciences, University of British Columbia, Vancouver, B.C., Canada, V6T 1Z3
[‡]Present address: Institute of Biological Chemistry, Washington State University, Pullman, WA 99164 USA

* Corresponding author: raymond.andersen@ubc.ca

Table S1. List of *Streptomyces* isolates, isolation media, coordinates of the collection site and their corresponding closely related type strains.
Table S2. Descriptions of the aerial and substrate mycelia, and soluble pigment
Table S3. NMR Data for Novobiocins **1**-**6** (600 MHz, DMSO-d_6)
Figure S1: ^{1}H NMR Spectrum of Desmethyldescarbamoylnovobiocin (**2**) recorded at 600 MHz in DMSO-d_6
Figure S2: ^{13}C NMR Spectrum of Desmethyldescarbamoylnovobiocin (**2**) recorded at 150 MHz in DMSO-d_6
Figure S3: ^{1}H NMR Spectrum of Desmethylnovobiocin (**3**) recorded at 600 MHz in DMSO-d_6
Figure S4: ^{13}C NMR Spectrum of Desmethylnovobiocin (**3**) recorded at 150 MHz in DMSO-d_6
Figure S5: ^{1}H NMR Spectrum of 4-Hydroxynovobiocin (**4**) recorded at 600 MHz in DMSO-d_6
Figure S6: ^{13}C NMR Spectrum of 4-Hydroxynovobiocin (**4**) recorded at 150 MHz in DMSO-d_6
Figure S7: ^{1}H NMR Spectrum of Desmethyldescarbamoyl-4-hydroxynovobiocin (**5**) recorded at 600 MHz in DMSO-d_6
Figure S8: ^{1}H NMR Spectrum of Desmethyl-4-hydroxynovobiocin (**6**) recorded at 600 MHz in DMSO-d_6
Figure S9: ^{13}C NMR Spectrum of Desmethyl-4-hydroxynovobiocin (**6**) recorded at 150 MHz in DMSO-d_6

Table S1. List of *Streptomyces* isolates and coordinates of the collection site

Strain (accession #)[a]	Isolation Medium	Collection site, depth (m)	Most closely related type strain[b]
RJA2895 (JX535235)	MM5	BF #6 48°52'N, 125°09'W, 82 m	*S. flavofungini* Szabo strain
RJA2910 (JX535236)	MM5	BF #5 48°52'N, 125°08'W, 80 m	*S. hawaiiensis* NRRL 15010
RJA2921 (JX535237)	MM8	BF #8 48°50'N, 125°13'W, 65 m	*S. deccanesis* DAS-139T
RJA2926 (JX535238)	MM5	BF #8 48°50'N, 125°13'W, 65 m	*S. sporoclivatus* LMG 20312
RJA2960	MM5	IA#7 49°25'N, 122°51'W, 95 m	*S. flavofungini* Szabo strain
RJA2961 (JF719041)	MM5	IA#12 49°26'N, 122°57'W, 65 m	*S. caeruleus* QD13II
RJA2969 (JX535239)	MM5	IA#12 49°26'N, 122°57'W, 65 m	*S. sampsonii* ATCC 25495
RJA3024	MM5	IA#19 49°24'N, 122°52'W, 35 m	*S. flavofungini* Szabo strain
RJA3025(JX535240)	MM3	IA #1 new mud missile lost in process of collecting	*S. rutgersensis* DSM 40077T
RJA3040	MM19	IA#19 49°24'N, 122°52'W, 35 m	*S. sampsonii* ATCC 25495
RJA3067	MM13	IA#1 new mud missile lost in process of collecting	*S. rutgersensis* DSM 40077T
RJA3074 (JX535241)	MM7	BF #7 48°52'N, 125°09'W, 82 m	*S. griseus* strain 52-1
RJA3265	MM10	BF #8 48°50'N, 125°13'W, 65 m	*S. sampsonii* ATCC 25495
RJA3407	MM39	HS#2 49°23'N, 123°15'W, 30 m	*S. flavofungini* Szabo strain
RJA3410	MM38	HS#2 49°23'N, 123°15'W, 30 m	*S. flavofungini* Szabo strain
RJA3622	MM16	IA#15 49°26'N, 122°52'W, 23 m	*S. sampsonii* ATCC 25495
RJA3937	MM47	GS#1 49°31'N, 123°58'W, 146 m	*S. rutgersensis* DSM 40077T
RJA3939	MM47	GS#1 49° 31'N, 123° 58'W, 146 m	*S. flavofungini* Szabo strain
RJA3948(JX535242)	MM47	GS#1 49° 31'N, 123°58'W, 146 m	*S. koyangensis* VK-A60
RJA3953	MM47	GS#1 49° 31'N, 123°58'W, 146 m	*S. sampsonii* ATCC 25495
RJA3956	MM5	GS#1 49° 31'N, 123°58'W, 146 m	*S. rutgersensis* DSM 40077T
RJA3957	MM5	GS#1 49° 31'N, 123°58'W, 146 m	*S. rutgersensis* DSM 40077T
RJA3958	MM5	GS#1 49° 31'N, 123°58'W, 146 m	*S. flavofungini* Szabo strain
RJA3972 (JF837443)	MM5	GS#1 49° 31'N, 123°58'W, 146 m	*S. rubrogriseus* LMG20318
RJA3973	MM5	GS#1 49° 31'N, 123°58'W, 146 m	*S. flavofungini* Szabo strain
RJA3974	MM5	GS#1 49° 31'N, 123°58'W, 146 m	*S. flavofungini* Szabo strain
RJA3980	MM5	GS#1 49° 31'N, 123°58'W, 146 m	*S. rutgersensis* DSM 40077T
RJA3983 (JX535243)	MM5	GS#1 49° 31'N, 123°58'W, 146 m	*S. violaceusniger* Tu 4113
RJA3990 (JF837445)	MM49	GS#1 49° 31'N, 123°58'W, 146 m	*S. speibonae* PK-Blue
RJA3995	MM48	GS#1 49° 31'N, 123°58'W, 146 m	*S. sampsonii* ATCC 25495
RJA3996	MM48	GS#1 49° 31'N, 123°58'W, 146 m	*S. flavofungini* Szabo strain
RJA3999	MM50	GS#1 49° 31'N, 123°58'W, 146 m	*S. sampsonii* ATCC 25495
RJA4019	MM5	GS#6 49°28'N, 123°55'W, 158 m	*S. flavofungini* Szabo strain
RJA4020 (JF510466)	MM5	GS#6 49°28'N, 123°55'W, 158 m	*S. caeruleus* QD13II
RJA4028	MM50	GS#6 49°28'N, 123°55'W, 158 m	*S. sampsonii* ATCC 25495
RJA4037	MM49	GS#12 49°21'N, 123°26'W, 70 m	*S. sampsonii* ATCC 25495
RJA4038(JX535244)	MM49	GS#5 49°28'N, 123°56'W, 143 m	*S. flocculus* NBRC 13041
RJA4040	MM47	GS#5 49°28'N, 123°56'W, 143 m	*S. rutgersensis* DSM 40077T
RJA4053	MM47	GS#12 49°21'N, 123°26'W, 70 m	*S. flavofungini* Szabo strain
RJA4054	MM47	GS#3 49°31'N, 123° 59'W, 102 m	*S. sampsonii* ATCC 25495
RJA4055(JX535245)	MM47	GS#3 49°31'N, 123° 59'W, 102 m	*S. drozdowiczii* NRRL B-24297
RJA4056	MM47	GS#3 49°31'N, 123° 59'W, 102 m	*S. rutgersensis* DSM 40077T
RJA4060	MM47	GS#6 49°28'N, 123°55'W, 158 m	*S. sampsonii* ATCC 25495
RJA4068 (JX535246)	MM47	HS#1 49°23'N, 123°15'W, 15 m	*S. anulatus* strain Malaysia
RJA4077	MM17	HS#2 49°23'N, 123°15'W, 30 m	*S. flavofungini* Szabo strain
RJA4079	MM17	HS#2 49°23'N, 123°15'W, 30 m	*S. flavofungini* Szabo strain
RJA4081	MM1	GS#11 49°23'N, 123°25'W, 70 m	*S. flavofungini* Szabo strain

BF = Bamfield; IA = Indian Arm; GS = Georgia Strait; HS = Howe Sound

[a]NCBI accession number.

[b]The corresponding type of strain had a level of similarity greater than or equal to 98.5%.

Table S2. Descriptions of the aerial and substrate mycelia, and soluble pigment

Strain	Collection site, depth (m)	Color on MM1 agar		
		**Aerial spore mass**	**Substrate mycelium**	**Soluble pigment**
RJA2895	BF #6, 82 m	medium-grey	deep brown	reddish- brown
RJA2910	BF #5, 80 m	white	black	reddish- brown
RJA2921	BF #8, 65 m	white	yellow	none
RJA2926	BF #8, 65 m	grey	white	none
RJA2960	IA #7, 95 m	yellow-pink	light yellow	none
RJA2961	IA #12, 65 m	yellow-pink	white	none
RJA2969	IA #12, 65 m	yellow-pink	dark brown	reddish- brown
RJA3024	IA #19, 35 m	yellow-pink	light yellow	none
RJA3025	IA #1	yellow-pink	light yellow	none
RJA3040	IA #19, 35 m	white	light yellow	none
RJA3067	IA #1	medium grey	dark brown	reddish- brown
RJA3074	BF #7, 82 m	yellow	yellow-pink	yellow
RJA3265	BF #8, 65 m	white	yellow	none
RJA3407	HS #2, 30 m	yellow	yellow	none
RJA3410	HS #2, 30 m	yellow-pink	light orange	none
RJA3622	IA #15, 23 m	yellow pink	dark brown	reddish- brown
RJA3937	GS #1, 146 m	yellow pink	reddish brown	brown
RJA3939	GS #1, 146 m	yellow-pink	reddish brown	brown
RJA3948	GS #1, 146 m	yellow	light brown	none
RJA3953	GS #1, 146 m	grey	dark brown	reddish- brown
RJA3956	GS #1, 146 m	white	white	none
RJA3957	GS #1, 146 m	grey	dark brown	reddish- brown
RJA3958	GS #1, 146 m	yellow-pink	dark brown	reddish- brown
RJA3972	GS #1, 146 m	grey	dark brown	reddish- brown
RJA3973	GS #1, 146 m	light-pink	dark brown	reddish- brown
RJA3974	GS #1, 146 m	light-pink	dark brown	reddish- brown
RJA3980	GS #1, 146 m	light-pink	dark brown	reddish- brown
RJA3983	GS #1, 146 m	white	light yellow	none
RJA3990	GS #1, 146 m	white	light yellow	none
RJA3995	GS #1, 146 m	light-pink	dark brown	reddish- brown
RJA3996	GS #1, 146 m	white	yellow-pink	none
RJA3999	GS #1, 146 m	grey	dark brown	reddish- brown
RJA4019	GS #6, 158 m	yellow-pink	dark brown	reddish- brown
RJA4020	GS #6, 158 m	grey	dark brown	reddish- brown
RJA4028	GS #6, 158 m	yellow-pink	dark brown	reddish- brown
RJA4037	GS #12, 70 m	yellow-pink	dark brown	reddish- brown
RJA4038	GS #5, 143 m	white	light brown	none
RJA4040	GS #5, 143 m	yellow-pink	yellow-pink	none
RJA4053	GS #12, 70 m	yellow-pink	Light brown	none
RJA4054	GS #3, 102 m	yellow-pink	Dark brown	reddish- brown
RJA4055	GS #3, 102 m	white	yellow-pink	none
RJA4056	GS #3, 102 m	white	yellow-pink	none
RJA4060	GS #6, 158 m	yellow-pink	dark brown	reddish- brown
RJA4068	HS #1, 15 m	medium grey	light brown	light amber
RJA4077	HS #2, 30 m	yellow-pink	light brown	Light amber
RJA4079	HS #2, 30 m	yellow-pink	dark brown	reddish- brown
RJA4081	GS #11, 70 m	yellow-pink	dark brown	reddish- brown

BF = Bamfield; IA = Indian Arm; GS = Georgia Strait; HS = Howe Sound

Table S3. NMR Data for Novobiocins **1-6** (600 MHz, DMSO-d_6).[a]

Atom #	δ_C/δ_N[b] 1	2	3	4	5	6	δ_H (*J* in Hz) 1	2	3	4	5	6
1	124.2	124.3	124.2	123.7	no	123.7						
2	129.8	129.8	129.8	120.6	120.6	120.6	7.74, s	7.73, s	7.74, s	7.30, s	7.30, s	7.30, s
3	127.2	127.2	127.2	127.5	127.5	127.5						
4	158.3	158.2	158.3	146.7	146.4	146.7						
5	114.2	114.2	114.2	144.3	144.3	144.3						
6	127.4	127.4	127.4	113.1	113.0	113.1	7.71, d (8.2)	7.70, d (8.4)	7.71, d (8.4)	7.29, s	7.29, s	7.29, s
7	28.1	28.1	28.1	28.3	28.3	28.3	3.26, d (7.2)	3.26, d (7.2)	3.26, d (7.3)	3.26, d (7.0)	3.26, d (7.3)	3.26, d (7.2)
8	122.6	122.6	122.6	122.8	122.8	122.8	5.30, bt (7.2)	5.30, bt (7.2)	5.30, bt (7.3)	5.30, bt (7.0)	5.30, bt (7.3)	5.30, bt, (7.2)
9	131.5	131.4	131.5	131.1	131.2	131.1						
10	17.7	17.7	17.7	17.7	17.7	17.7	1.69, bs	1.69, bs	1.69, bs	1.69, bs	1.69, bs	1.70, s
11	25.6	25.6	25.6	25.6	25.6	25.6	1.69, bs	1.69, bs	1.69, bs	1.68, bs	1.68, bs	1.68, bs
12	166.5	166.4	166.5	166.7	166.5	166.7						
4-OH							10.05, s	10.03, s	10.05, s	8.91, s	8.90, s	8.91, s
R (H/OH)							6.85, d (8.2)	6.84, d (8.4)	6.85, d (8.4)	9.61, s	9.60, s	9.61, s
1’	160.6	160.8	160.7	160.6	no	160.6						
2’	101.5	no	101.4	101.7	no	101.6						
3’	159.3	no	159.3	158.9	no	158.9						
4’	121.8	121.8	121.8	121.8	121.8	121.8	7.73, d (8.9)	7.71, d (8.4)	7.73, d (9.1)	7.73, d (9.1)	7.71, d (8.6)	7.73, d (9.0)
5’	110.03[1]	109.9	110.04	110.0	110.0	110.0	7.16, d (8.9)	7.15, d (8.4)	7.16, d (9.1)	7.16, d (9.1)	7.15, nd[c]	7.16, d (9.0)
6’	156.9	157.2	157.1	156.9	157.5	157.1						
7’	112.8	112.5	112.8	112.8	112.5	112.8						
8’	150.6	150.6	150.6	150.5	150.6	150.5						
9’	110.05[1]	no	109.96	110.8	no	110.0						
10’	8.3	8.4	8.32	8.3	8.3	8.3	2.21, s	2.19, s	2.22, s	2.21, s	2.19, s	2.22, s
2’-NH	-274.1	no	-274.6	-272.9	no	-273.0	9.23, s	9.17, bs	9.23, s	9.18, s	9.15, bs	9.17, bs
3’-OH							11.96, s	11.95, s	11.95, s	12.06, s	12.04, s	12.05, s
1”	98.4	98.5	98.6	98.5	98.5	98.6	5.53, d (2.7)	5.52, d (1.4)	5.52, d (2.3)	5.53, d (2.0)	5.52, bs	5.52, d (2.3)
2”	68.7	70.4	68.3	68.7	70.4	68.3	4.07, t (2.7)	3.92, m	4.07, m	4.07, m	3.92, m	4.07, m
3”	70.3	67.5	70.4	70.3	67.5	70.4	5.15, dd (10.0, 2.7)	3.89, m	5.05, dd (9.9, 3.1)	5.15, dd (9.4, 3.0)	3.89, m	5.05, dd (9.8, 3.2)
4”	80.7	72.3	69.8	80.7	72.3	69.8	3.47, d (10.0)	3.57, dd (9.1, 5.4)	3.75, d (9.9)	3.47, d(9.4)	3.57, dd (9.1, 5.4)	3.75, dd (9.8, 6.2)
5”	78.1	78.5	78.9	78.1	78.3	78.9						
6”	22.7	22.5	22.7	22.7	22.5	22.7	1.04, s	1.01, s	1.06, s	1.04, s	1.01, s	1.05, s
7”	28.4	28.6	28.4	28.4	28.6	28.4	1.26, s	1.21, s	1.23, s	1.26, s	1.21, s	1.23, s
2”-OH							5.60, bs	5.16, d (4.4)	no	5.59, bs	5.16, d (4.4)	5.45, d (5.1)
R’ (-C(O)NH$_2$/OH)	156.3		156.6	156.3		156.6						
R’ (-C(O)NH$_2$/OH)	-302.6		-302.9	-302.9		-302.5	6.73, bs/6.52, bs	4.79, d (5.4)	6.50, bs/6,50, bs	6.73, bs/6.52, bs	4.79, d (5.4)	6.49, bs/6.49, bs
R” (Me/OH)	61.0			61.0			3.46, s	4.96, d (5.4)	no	3.46, s	4.96, d (5.4)	5.14, d (6.2)

[a]Numbering according to reference 43.

[b]The ^{15}N assignments were not calibrated with an external standard. The δ value has an accuracy of about 1 ppm in reference to CH_3NO_2 (0 ppm) and are assigned on the basis of ^{15}NHSQC and ^{15}NlrHMQC correlations.

[c]Multiplicity not determined due to overlapping of signal with the residual TFA signal.

[1]Assignments within a column are interchangeable.

no – not observed.

Figure S1: ^{1}H NMR Spectrum of Desmethyldescarbamoylnovobiocin (**2**) recorded at 600 MHz in DMSO-d_6

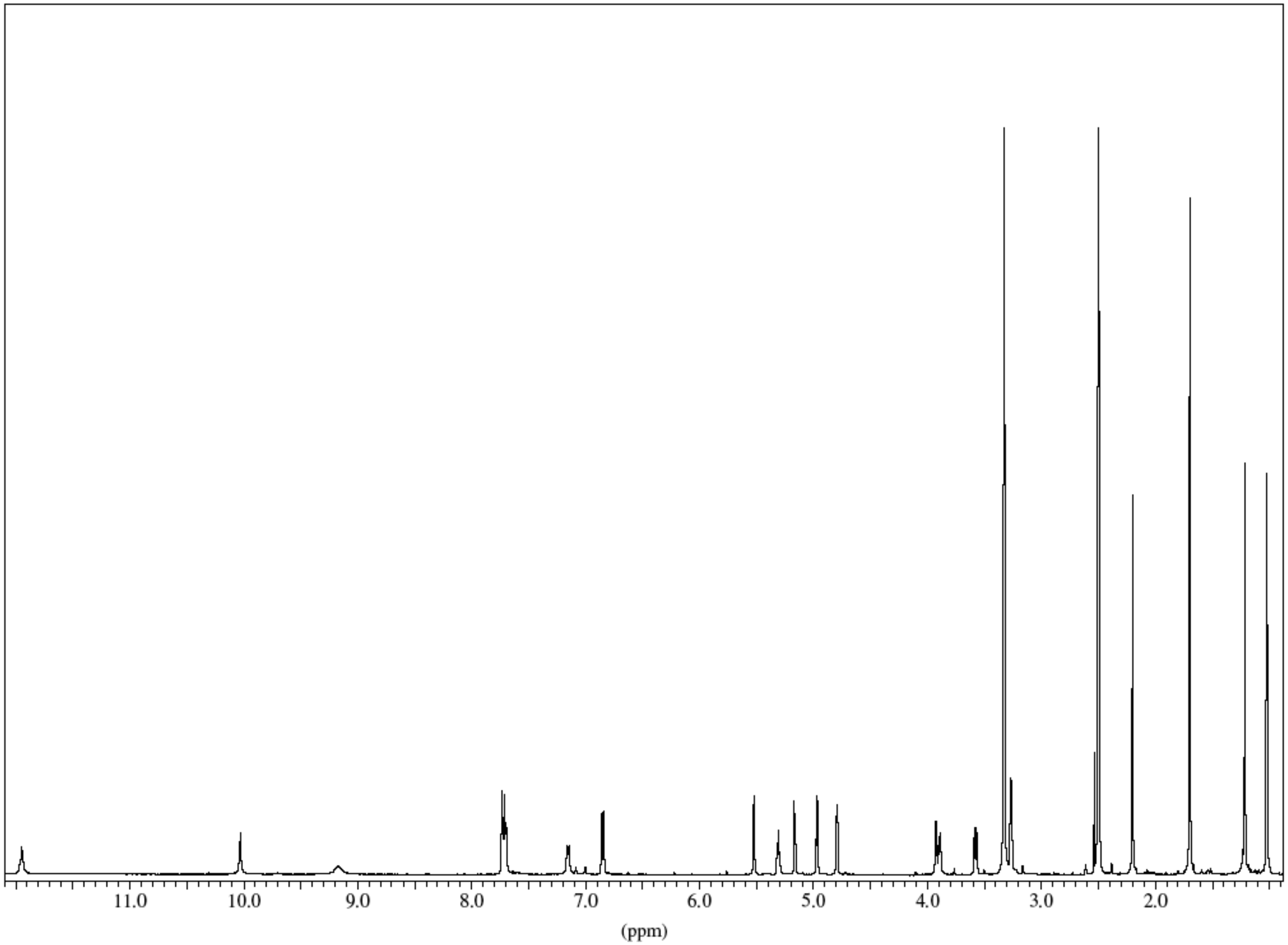

Figure S2: ^{13}C NMR Spectrum of Desmethyldescarbamoylnovobiocin (**2**) recorded at 150 MHz in DMSO-d_6

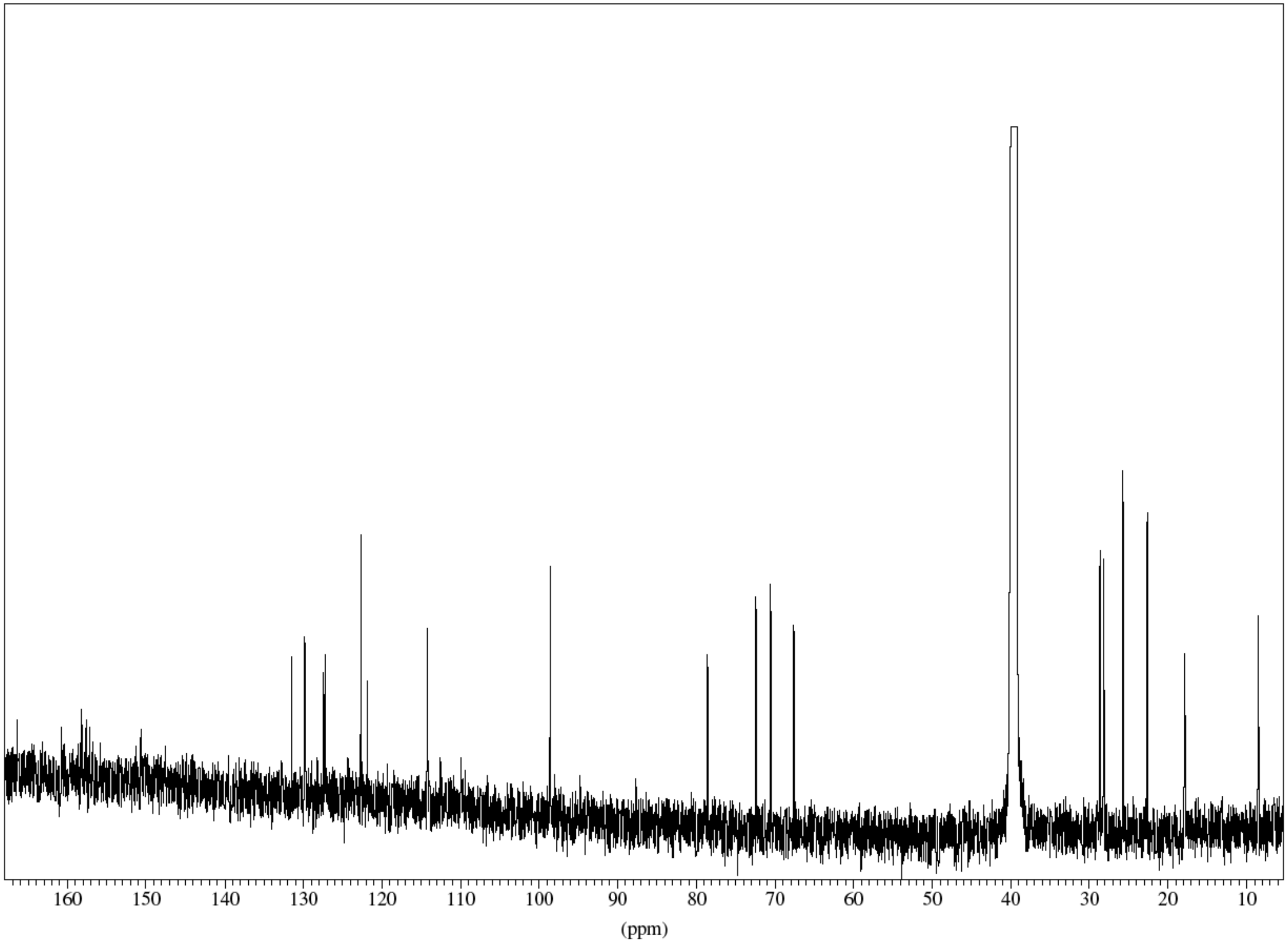

Figure S3: ^{1}H NMR Spectrum of Desmethylnovobiocin (**3**) recorded at 600 MHz in DMSO-d_6

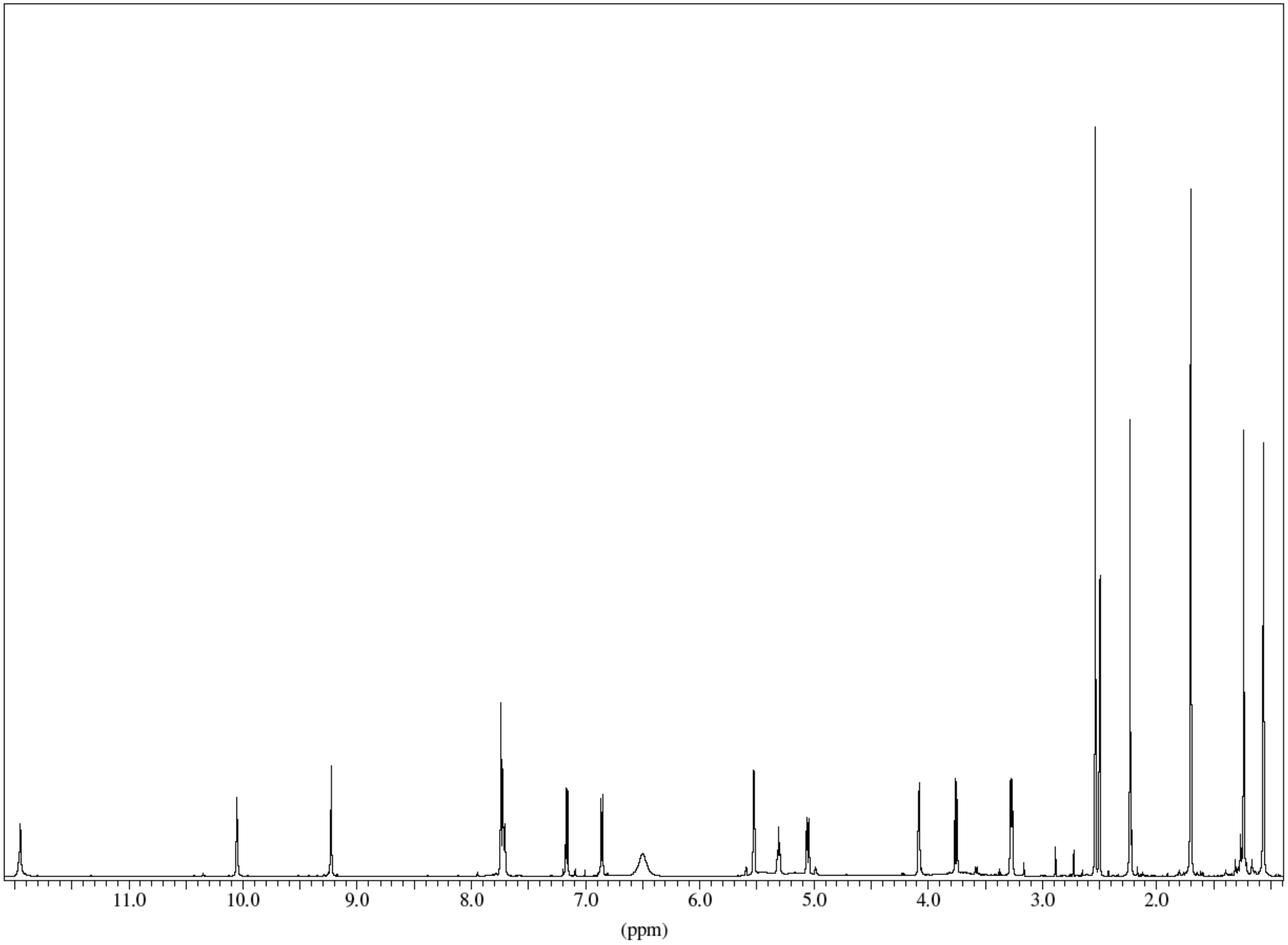

Figure S4: ^{13}C NMR Spectrum of Desmethylnovobiocin (**3**) recorded at 150 MHz in DMSO-d_6

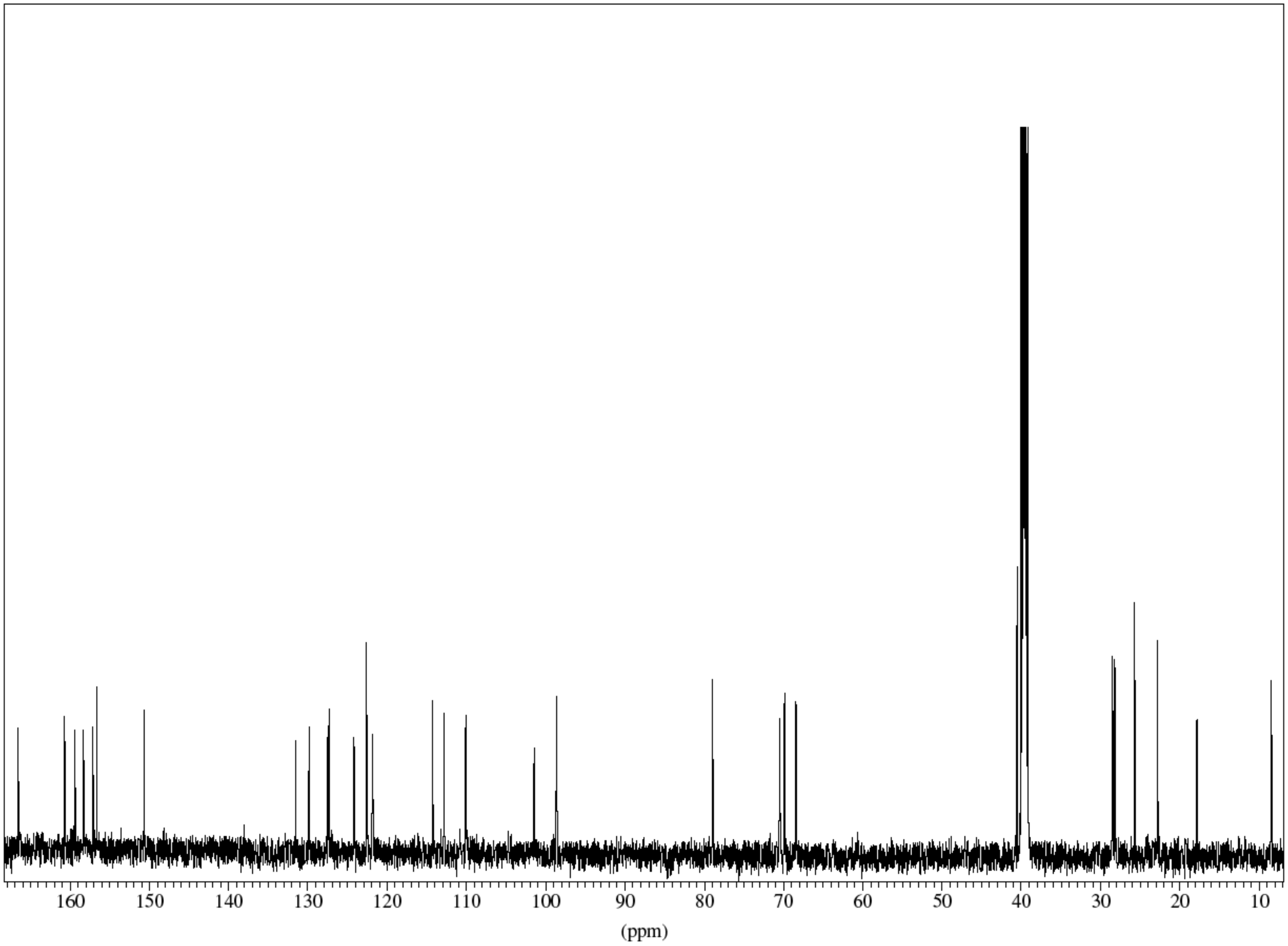

Figure S5: ^{1}H NMR Spectrum of 4-Hydroxynovobiocin (**4**) recorded at 600 MHz in DMSO-d_6

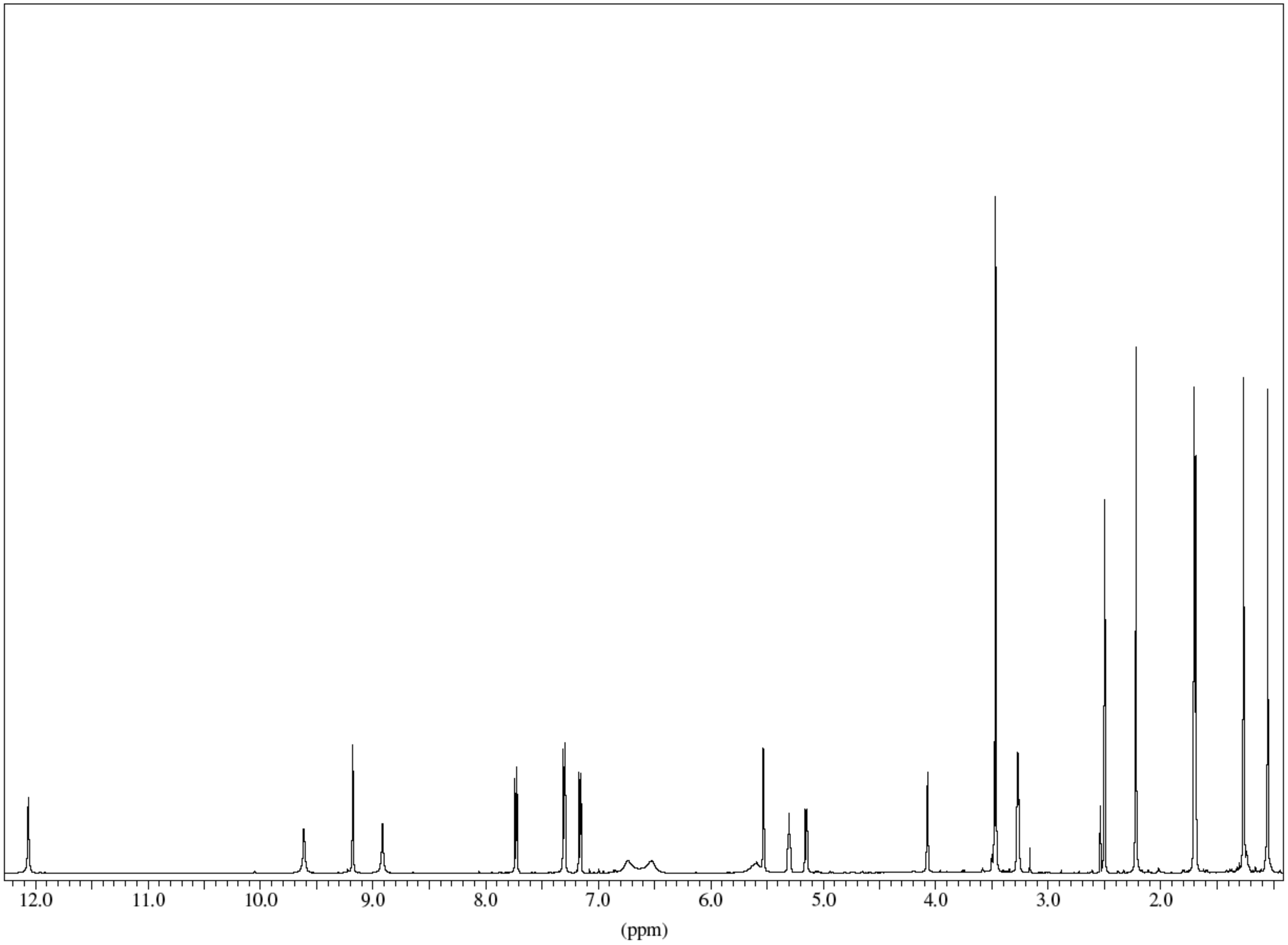

Figure S6: ^{13}C NMR Spectrum of 4-Hydroxynovobiocin (**4**) recorded at 150 MHz in DMSO-d_6

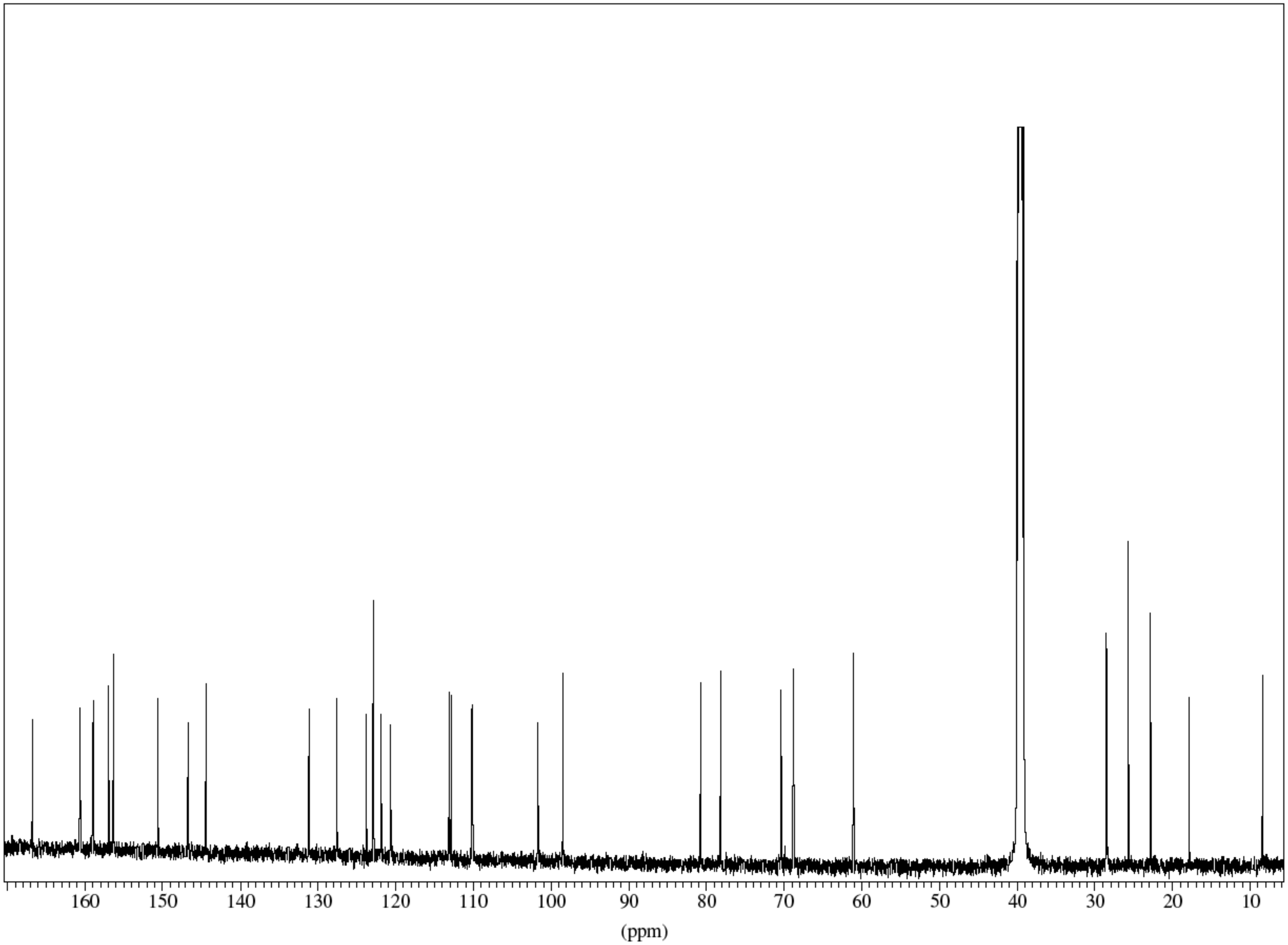

Figure S7: ^{1}H NMR Spectrum of Desmethyldescarbamoyl-4-hydroxynovobiocin (**5**) recorded at 600 MHz in DMSO-d_6

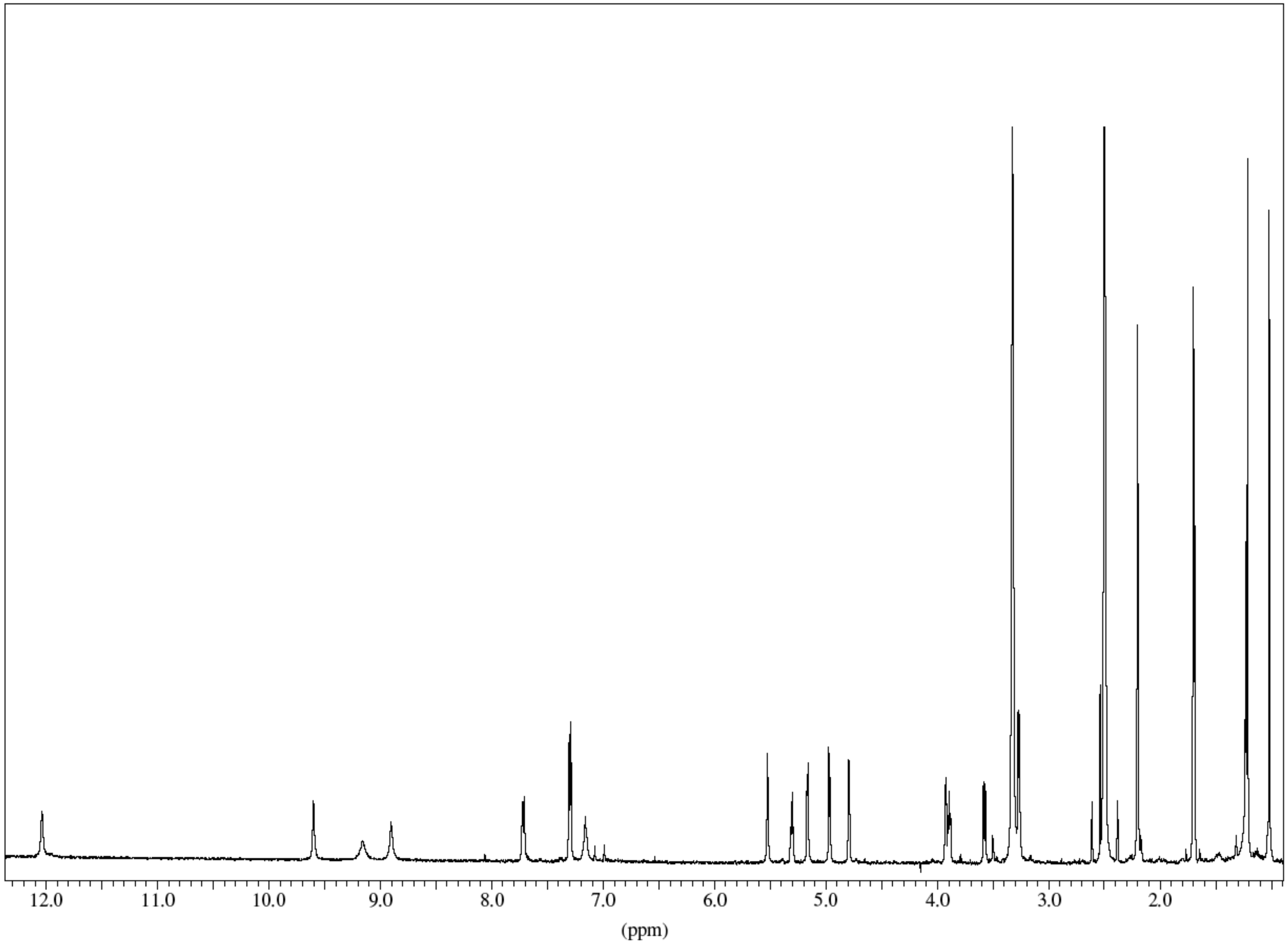

Figure S8: ^{1}H NMR Spectrum of Desmethyl-4-hydroxynovobiocin (**6**) recorded at 600 MHz in DMSO-d_6

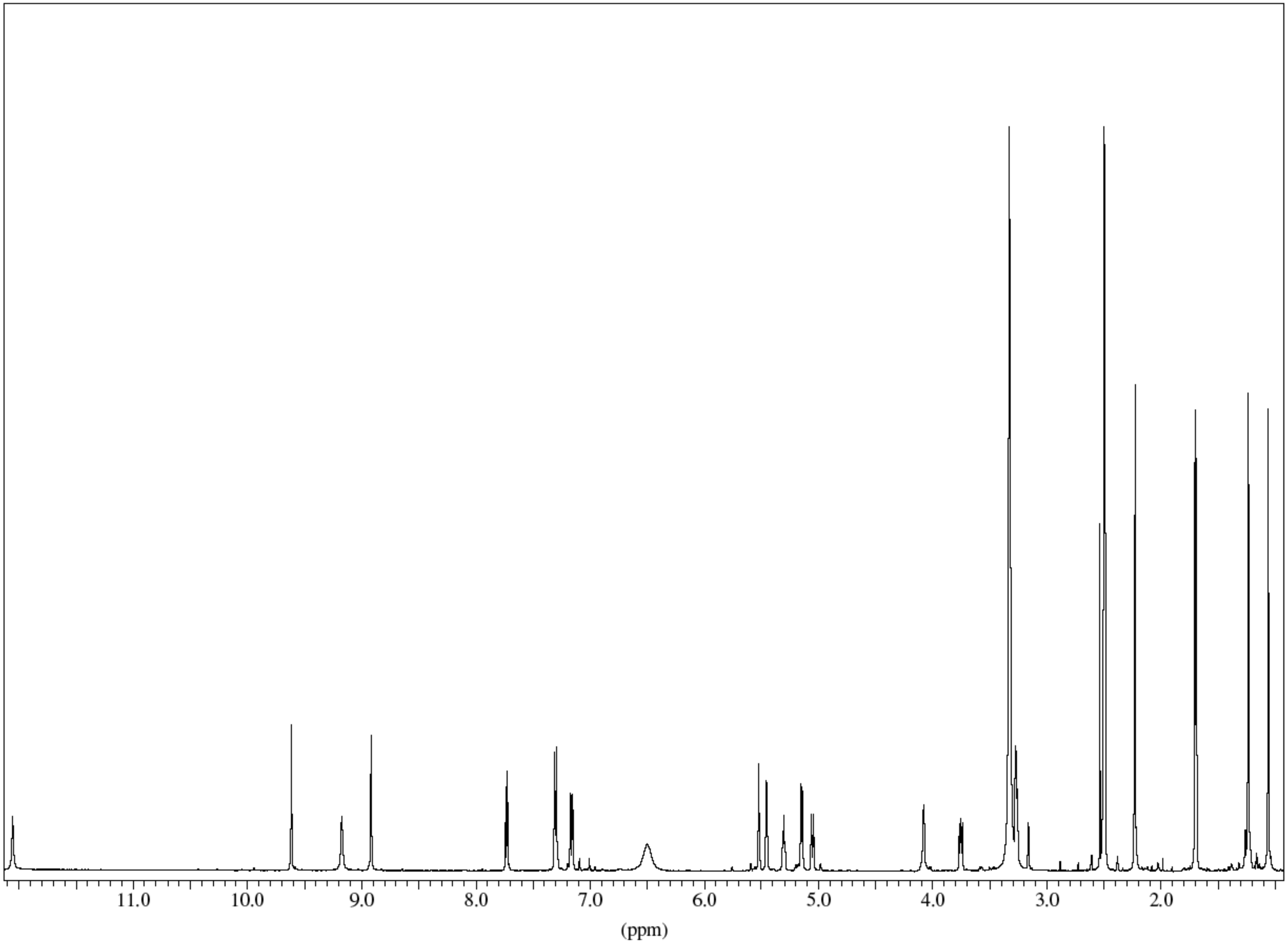

Figure S9: ^{13}C NMR Spectrum of Desmethyl-4-hydroxynovobiocin (**6**) recorded at 150 MHz in DMSO-d_6

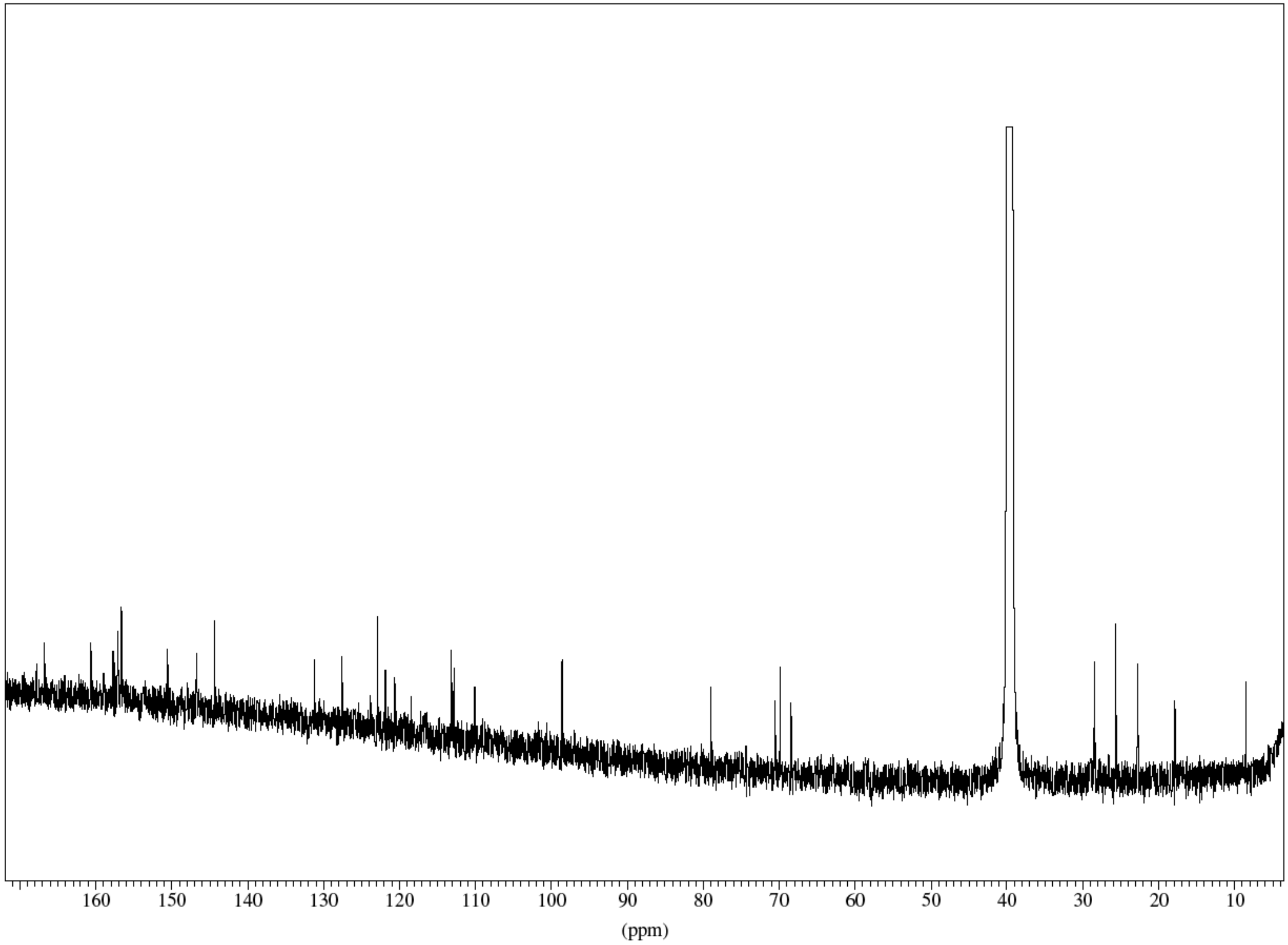

Supplement: File S1 — Table S1. List of Streptomyces isolates and coordinates of the collection site. Table S2. Descriptions of the aerial and substrate mycelia, and soluble pigment. Table S3. NMR Data for Novobiocins 1-6 (600 MHz, DMSO-d 6). Figure S1: 1H NMR Spectrum of Desmethyldescarbamoylnovobiocin (2) recorded at 600 MHz in DMSO-d 6. Figure S2: 13C NMR Spectrum of Desmethyldescarbamoylnovobiocin (2) recorded at 150 MHz in DMSO-d 6. Figure S3: 1H NMR Spectrum of Desmethylnovobiocin (3) recorded at 600 MHz in DMSO-d 6. Figure S4: 13C NMR Spectrum of Desmethylnovobiocin (3) recorded at 150 MHz in DMSO-d 6. Figure S5: 1H NMR Spectrum of 4-Hydroxynovobiocin (4) recorded at 600 MHz in DMSO-d 6. Figure S6: 13C NMR Spectrum of 4-Hydroxynovobiocin (4) recorded at 150 MHz in DMSO-d 6. Figure S7: 1H NMR Spectrum of Desmethyldescarbamoyl-4-hydroxynovobiocin (5) recorded at 600 MHz in DMSO-d 6. Figure S8: 1H NMR Spectrum of Desmethyl-4-hydroxynovobiocin (6) recorded at 600 MHz in DMSO-d 6. Figure S9: 13C NMR Spectrum of Desmethyl-4-hydroxynovobiocin (6) recorded at 150 MHz in DMSO-d 6. (PDF) [file pone.0077078.s001.pdf]
